# Semantic Relations Cause Interference in Spoken Language Comprehension When Using Repeated Definite References, Not Pronouns

**DOI:** 10.3389/fpsyg.2016.00214

**Published:** 2016-03-01

**Authors:** Sara A. Peters, Timothy W. Boiteau, Amit Almor

**Affiliations:** ^1^Social and Behavioral Sciences Department, Newberry CollegeNewberry, SC, USA; ^2^Department of Psychology, University of South CarolinaColumbia, SC, USA; ^3^Linguistics Department, University of South CarolinaColumbia, SC, USA

**Keywords:** reference, repeated name penalty, pronouns, semantic relations, spoken language comprehension

## Abstract

The choice and processing of referential expressions depend on the referents' status within the discourse, such that pronouns are generally preferred over full repetitive references when the referent is salient. Here we report two visual-world experiments showing that: (1) in spoken language comprehension, this preference is reflected in delayed fixations to referents mentioned after repeated definite references compared with after pronouns; (2) repeated references are processed differently than new references; (3) long-term semantic memory representations affect the processing of pronouns and repeated names differently. Overall, these results support the role of semantic discourse representation in referential processing and reveal important details about how pronouns and full repeated references are processed in the context of these representations. The results suggest the need for modifications to current theoretical accounts of reference processing such as Discourse Prominence Theory and the Informational Load Hypothesis.

## Introduction

Coherent discourse can be established via different forms of repeated reference to the same referent, such as repeated names (e.g., *Jane*), definite descriptions (e.g., *the girl*), and pronouns (e.g., *she, her*). The form used for repeated references is generally related to the discourse status of the referent (Almor and Nair, 2007). For example, full definite descriptions are often used to introduce referents that were not previously mentioned or to refer to referents that were previously mentioned but are not currently salient in the discourse (Gundel et al., 1993). In contrast, pronouns are often and naturally used to refer to previously mentioned referents that are salient in the context of the discourse (Ariel, 1990; Gundel et al., 1993). One of the clearest empirical demonstrations of the relationship between referential form and referents' discourse status is the repeated name penalty (RNP). This effect was first demonstrated as the slower reading of repeated proper names than pronouns when referring to the most salient referent in the discourse (Gordon et al., 1993). The RNP was later extended to repeated definite references, which are read slower when the referent is focused than when it is not (Almor, 1999). Multiple theories have been developed to explain the relation between reference form and the discourse status of referents (for a review see Almor and Nair, 2007). Although many of these theories explain this relation on the basis of general language and memory mechanisms that are not modality specific, much of the relevant empirical findings are based on reading paradigms. One aim of the present work was therefore to test whether the RNP occurs in spoken language comprehension, and if so, to test the predictions of existing theories about the timing and presence of the memory processes that underlie this effect.

Gordon et al. (1993) originally explained the RNP in terms of Centering Theory (Grosz et al., 1995), which Gordon and Hendrick (1998) later developed into Discourse Prominence Theory (DPT). According to DPT, repeated full references are initially interpreted as introducing new discourse entities that later require integration with the representation of the previous discourse, while pronouns are initially interpreted as co-referential, and thus do not generate new representations. In addition, referential processing is governed by a set of construction rules applied as part of building and maintaining a discourse representation. When a proper name is encountered, a construction rule generates a new representation in the discourse model, but when a pronoun is used, a different construction rule searches for a matching referent in the list of previously mentioned referents in decreasing order of prominence (Gordon and Hendrick, 1998). This model explains the RNP as reflecting the application of a special “equivalence” construction rule that reconciles the representation of the new referent generated by the proper name construction rule, and the representation of the referent already in memory. The rule searches the list of referents in ascending prominence order, thus taking longer to identify matches with prominent referents than with non-prominent referents. DPT therefore attributes the RNP to the time needed to merge the newly generated representation evoked by repeated full references with the existing discourse representation. Importantly, DPT also argues that repeated references are processed similarly to new references, at least during the initial stages of processing.

An alternative approach is the Informational Load Hypothesis (ILH; Almor, 1999, 2000, 2004; Almor and Nair, 2007), which attributes the RNP to memory interference between the representation of the referential expression and the existing representation of the referent in memory. In its original formulation (Almor, 1999, 2004), the theory emphasized the interaction between pragmatic principles and memory constraints but did not explicitly address the detailed processing time course of referential expressions. However, a more recent version of the theory (Almor and Nair, 2007) includes specific claims about the stages that are involved in processing referential expressions. According to this version, referential processing involves multiple stages that are differentially affected by the salience of the referent in the discourse. In Stage 1, the incoming referential expression undergoes lexical processing before it can be integrated into the discourse representation. This stage results in the activation of a representation of the referential expression that is initially separate from the prior representation of the discourse. Priming from a salient referent may facilitate this initial activation. Stage 1 in this view is compatible with Ledoux et al.'s (2007) finding of an initial stage of processing involving priming due solely to repetition, regardless of other co-referential processes. In Stage 2, this newly activated representation is integrated with the prior discourse representation. These two stages can overlap, but Stage 2 generally takes longer to complete than Stage 1.

Although both DPT and the ILH argue that processing definite reference results in the formation of a new representation, the two theories differ in their view of how and when this representation is processed. In DPT, repeated reference is processed just like a new reference. According to this theory, the difference between a new reference and a repeated one only occurs when a potential referent is not found through the serial search of current referents. Thus, in this account, repeated, and new references are processed similarly. In contrast, according to the ILH, the newly formed representation can interact with the existing discourse representation right from the start. In particular, the initial formation of the reference (Stage 1) can be facilitated by semantic overlap with an existing representation, but the integration of the representation with the discourse (Stage 2) is prone to interference due to semantic overlap. According to the ILH, repeated references are simply more likely to trigger these effects than new references, which do not necessarily overlap semantically with existing representations (Almor and Nair, 2007).

According to the ILH, maintaining similar, yet distinct representations results in interference until integration of the new representation is complete. This interference reflects the effort associated with maintaining simultaneously activated representations in a limited-capacity memory system. Therefore, the extent of this interference is affected by the activation of the referent in memory, such that salient referents can cause more interference during integration than less salient ones. This interference is also affected by the semantic overlap between the representations of the referential expression and the existing representation of the referent in memory. When the referent is already salient in the discourse, high overlap between the two may result in greater interference. In this view, pronouns minimize memory interference during integration because they do not evoke a rich representation in memory. Pronouns are therefore generally preferred when the referent is salient and can be easily identified. Thus, according to both the ILH and DPT, repeated full reference evokes an initial representation that is separate from the existing representation of the referent in memory. However, while DPT considers this representation to be equivalent to that of a new reference, the ILH considers it as an initial lexical representation that could lead to memory interference during integrative processing. Specifically, Almor (1999) argued that a high level of semantic similarity between a referential expression and the memory representations of previously mentioned referents makes it harder to maintain the representation of the discourse in a working memory mechanism specializing in the manipulation of semantic information. Almor based this argument on an analogy to the detrimental effect phonological similarity has on word list recall (Baddeley, 1992, 1996). This analogy assumes that the representation of discourse referents relies on a limited capacity memory system of semantic representations and that referential processing activates and manipulates these representations.

Cowles et al. (2010) presented evidence against this analogy. Using a word recall task modeled after the original Baddeley experiments, Cowles et al. replicated Baddeley's original finding of memory interference associated with phonological overlap between words in the memory list, but found no evidence for similar interference associated with semantic overlap. This suggests that the difficulty associated with semantic overlap during reference processing may reflect a memory process dissimilar from phonological interference in Baddeley's recall task. One phenomenon that has been linked with detrimental effects of semantic overlap on memory recall is the Cue-Overload effect (Watkins and Watkins, 1975), in which the efficiency of a retrieval cue is reduced when it has been used previously as a retrieval cue for a semantically similar but different item set. This likely reflects the activation of superfluous information, rendering the intended recall target less distinctive and therefore interfering with selection and processing.

A similar phenomenon may occur during reference processing and may help explain why semantic overlap can hinder the processing of referential expressions. In particular, it may be that a high level of semantic overlap between a referential expression and the existing representation of a referent increases the activation of the referent and the information already associated with it in long-term memory. This spreading activation occurs at the expense of processing other information in the discourse. Thus, semantic overlap and repetition initially result in facilitation; however, this facilitation results in the activation of other information in memory, and consequently in a reduced ability to process new discourse information until the integration of the referential expression is complete. This explanation is in fact more compatible with the vast literature on the facilitative memory effects of semantic overlap than the original explanation made in Almor (1999).

These theories appeal to general language and memory processes that are not specific to reading. However, most of the relevant research on the RNP has employed reading-based paradigms. In a study of reference processing in spoken language comprehension, Dahan et al. (2002) showed that when the referent is salient but not in focus, repeated definite references can be used felicitously without penalty. However, Dahan et al. only used stimuli in which the target references appeared in the grammatical object position of imperative instructions (e.g., “Put the X above the Y”). Thus, the study does not answer the question of whether the RNP occurs in spoken language comprehension. Another study that examined the RNP in spoken language comprehension is Almor and Eimas (2008), who found an initial facilitation in a lexical decision task but poor memory in a delayed recall task of information from discourses with repeated definite references to salient referents. Although this result suggests that the RNP extends to spoken language comprehension, it provides only limited information about the time course of the underlying processing (i.e., initial facilitation followed by delayed interference) or about the specifics of the underlying memory processes.

Given this previous work, the goals of the present study were to investigate the RNP in spoken language comprehension, examine the time course of reference processing in spoken language comprehension, and better understand the underlying memory processes and their influences. Experiment 1 examined whether an effect analogous to the RNP can be observed in spoken language comprehension using the visual world paradigm (VWP; Tanenhaus et al., 1995) and at the same time examine the time course and nature of the underlying memory processes in order to test the theories presented above. The visual displays used in the experiments were borrowed from Yee and Sedivy (2006) and contained items that were semantically related to one another within the displays. While these semantic relations were not exclusively based on shared category membership as in previous work on semantic overlap in reference processing (e.g., Almor, 1999), the inclusion of the semantically related items within Experiment 1 aimed to provide a more general test of activation of semantic information during reference processing and the RNP. Thus, this experiment aimed to test the following predictions of DTP and ILH in regards to reference processing and interference:

1. According to DPT (Gordon and Hendrick, 1998), repeated definite references are initially processed like new references. The similarity between the two forms is greatest when the referent is salient because the search for an existing referent in the discourse representation in the case of a full noun reference proceeds in increasing order of salience Thus, the referent search for a repeated definite reference to the most salient referent will require scanning the entire list of potential referents just as for a new definite reference.
2. According to the ILH (Almor, 1999), both pronouns and repeated references are initially interpreted as referring to a previously mentioned and salient referent. Although the early processing of repeated references could be facilitated due to repetition priming, memory interference during integrative processing should lead to delayed processing later on, especially if the reference was to a highly salient referent.
3. The two possible views of the underlying interference that were reviewed can also be tested in Experiment 1. If, in line with Almor (1999), semantic competitors within the display create interference, the resulting activation should resemble the semantic competitor effect observed by Yee and Sedivy (2006) during initial lexical processing. In contrast, interference that is generated by items that have been linked earlier in discourse with a salient referent, should be reflected in the activation of the information that was originally mentioned. This outcome would support the view that the interference underlying the RNP reflects the activation of information related to the referent at the expense of further processing the discourse.

Experiment 2 further tested the effect of explicitly mentioning a semantically related referent (e.g., *cat*) in previous discourse on the processing of a subsequent reference to a new target reference (e.g., *mouse*). In particular, this experiment aimed to determine whether such mention would have a facilitative or detrimental effect on processing a repeated reference to the target item later within the discourse. Specific predictions tested were as follows:

1. According to a cue-based retrieval explanation of reference processing, pre-existing semantic relations should facilitate the retrieval of the representation of the referent. If those referents have long term associations (semantic associations), the effect should be larger than if the association only was established in the discourse.
2. According to the ILH, the relationships (pre-existing or established in the discourse) should have a different effect on the processing of potential referents when repeated and pronoun references are used. The interference in processing repeated anaphors is expected to be greater with long-term relations in place between the referents than when the relationship between the referents has only been established in the discourse. This is hypothesized to be due to the increased activation of a related previously mentioned item than an unrelated previously mentioned item.

## Experiment 1

We used the VWP in order to obtain detailed information about the time course of activation of potential referents. This paradigm has been previously used to study referential processing (e.g., Dahan et al., 2002) but not for examining the RNP. Listeners' gazes in the VWP are known to be closely time locked to the unfolding language input (e.g., Allopenna et al., 1998; Arnold et al., 2004). Additionally, while previous research has shown that characteristics of the display influence fixation patterns, here we control properties of the referents in the visual display and allow for a preview time before each item begins to minimize these effects, as explained further in the Materials Section. Therefore, although the theories we test here were not previously tested using the VWP, we assume, following other research on the VWP, that the effects of language on attention and eye movements are immediate and reliable. Furthermore, we assume that the eye movements reflect the processing of and attention to the *specific* on-screen referent currently fixated (Tanenhaus et al., 1995). Thus, if interference in reference resolution is caused by working memory processes, those delays should also be reflected in the eye-movement record, and the delay can be evaluated in the models used to compare fixations.

**Figure 1:**
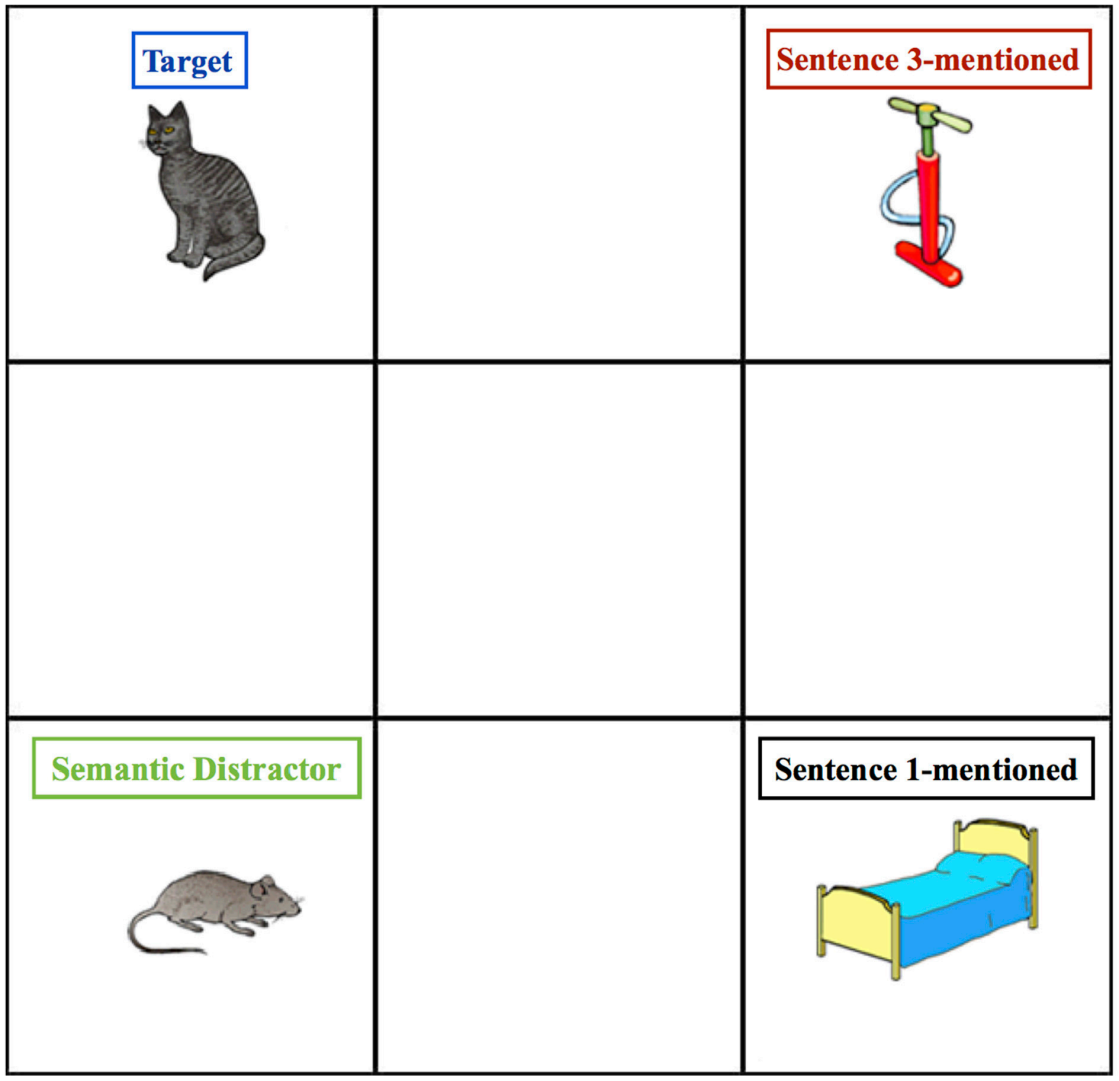
**Sample visual display, labeled**. Colors correspond to data graphed in future figures.

We constructed 3-sentence discourses that made a target referent salient in two ways: the target referent was mentioned in the grammatical subject position of two sentences prior to the critical sentence (Sentence 3), and it was referred to with a pronoun in the second of these sentences. Both manipulations have been previously shown to effectively increase salience and lead to the RNP (Gordon et al., 1993). In the critical sentences, we contrasted repeated definite references (the *Repeated* condition), pronoun references (the *Pronoun* condition), and definite references to new referents (the *New* condition). A separate study confirmed that these items elicit the RNP (Peters and Almor, 2006) in a text-based, self-paced reading paradigm that also served to pilot the items for the current work. In a critical experiment in that study, participants read discourses that were almost identical to the present items and took longer to read the third sentence in the Repeated condition than in the Pronoun condition. Another experiment in the study using the same items but without the second sentence, found that participants took longer to read the final sentence in the Pronoun condition than the Repeated condition. The second experiment thus showed that the RNP found in the first experiment was not simply a result of baseline differences in reading times between the critical sentences in the different conditions. In the present study, we contrasted for each of the referential conditions (Pronoun, Repeated, and New) fixations to the target referent (*Target*), a potential referent that was semantically related to the target referent (*Semantic Distractor*), a referent that was not semantically related to the target but that was mentioned in Sentence 1 (*Sentence 1-mentioned*), and an unrelated referent that was mentioned for the first time in the critical sentence (*Sentence 3-mentioned*). Figure 1 shows a sample experimental display and Table 1A shows the corresponding verbal stimulus in all three conditions. Detailed predictions derived from the theories presented in the introduction are stated below, in terms of fixations to the visual displays based on the discourses used in the experiment. An important aspect of this design is that since our critical sentences occurred several seconds and two sentences after the pictures were originally presented, eye movement patterns are unlikely to reflect any effects of the visual properties of the depicted objects (Henderson and Ferreira, 2004).

1. DPT argues that repeated references are initially processed as new references. Therefore, in the critical sentence (Sentence 3), listeners should initially interpret both the repeated and the new references as mentions of a new object, and look at an object not yet mentioned, which is the Semantic Distractor (*mouse*) in both conditions. This should result in comparable number and rate of increase in looks to the Semantic Distractor in the New and Repeated conditions following the critical reference. However, this should not be the case in the Pronoun condition, because identifying the referent of a pronoun proceeds in decreasing salience order, thus leading to the immediate identification of the Target (*cat*) as the intended referent.
2. According to the ILH, the initial processing of repeated references could be facilitated but the Repeated condition should lead to interference later in the critical sentence (Sentence 3) as integration takes place and the discourse unfolds. This should be reflected in an overall smaller number and a lower rate of increase in looks to the second referent mentioned (Sentence 3-mentioned, *pump*) in the critical sentence in the Repeated condition relative to the Pronoun condition. As the effect of the interference dissipates, this rate of fixation is likely to increase resulting in a larger quadratic component in the Repeated relative to the Pronoun condition.
3. In terms of interference types, more gazes, or a higher rate of fixations to the Semantic Distractor than to the Sentence 3-mentioned before it is heard, will indicate the activation of semantic representations. Such activation would be similar to that generated by initial lexical processing, in line with Almor (1999). In contrast, more gazes and a higher rate of fixations to the previously mentioned referent (Sentence 1-mentioned, *bed*) than to another referent that has not been mentioned (Semantic Distractor, Sentence 3-mentioned) would suggest that the interference is related to the activation of the information that was originally mentioned with the Target referent. This would support the cue-overload view that the interference underlying the RNP reflects the activation of information related to the referent at the expense of further processing the discourse.

**Table 1:** **Sample verbal item in all three conditions from (A) Experiment 1 and (B) Experiment 2**.

**Sentence #**	**Condition**	**Sentence**
**(A)**		
1		The cat is diagonal to the bed.
2		It is in the upper left corner.
3	*Pronoun*	It is next to the pump.
	*Repeated*	The cat is next to the pump.
	*New*	The pump is next to the cat.
**(B)**		
1	*Unrelated*	The cat is diagonal to the bed.
	*Related*	The cat is above the mouse.
2		It is in the upper left corner.
3	*Pronoun*	It is next to the pump.
	*Repeated*	The cat is next to the pump.

*Sentence 1 and 3 varied by condition, Sentence 2 remained constant. Participants heard one combination of each item, and each participant heard 6 of each combination of Unrelated-Pronoun, Unrelated-Repeated, Related-Pronoun, Related-Repeated within the experiment. Sentences 1 and 2 were the same in all conditions, while Sentence 3 varied by condition. (B) Sample verbal item in Experiment 2 in all conditions*.

As previously noted, the VWP provides finely tuned time course information about the activation and processing of the information presented in the display. In order to fully utilize this information, we modeled the fixation results using growth curve analyses (GCA) (Mirman et al., 2008). As we explain below, GCA allows us to test predictions about both the number and rate of change of looks in the different conditions. Importantly, this type of analysis allows us to explore the dynamics of fixation changes as well as sustained attention to a referent in a time window of interest. This would have been impossible under the common approach of averaging fixations in separate (and typically large) time windows. To the best of our knowledge, the use of GCA to study these dynamics of reference processing is novel. To reduce concerns related to the dependence between fixations to the different pictures, the majority of our analyses compared fixations to a particular display item across discourse conditions (e.g., fixations to Semantic Distractor in Pronoun vs. Repeated conditions). However, when the critical theoretical prediction rests on the difference in looks to different display items, we also included an analysis comparing fixations to different display items.

## Method

### Participants

Forty-nine undergraduate students recruited from the University of South Carolina Psychology Department's participant pool participated in this experiment for course credit. All participants provided informed consent in accordance with the University's IRB. All participants were native speakers of American English.

### Materials

As shown in Figure 1 and Table 1A, each item consisted of a pictorial display showing four objects arranged in the corners of a 3 × 3 grid, and a corresponding 3-sentence discourse. The pictorial displays were taken from Yee and Sedivy (2006) and included 24 experimental, 48 filler, and 4 practice displays. Experimental displays showed 2 semantically related objects [e.g., a Target (*cat*) and a Semantic Distractor (*mouse*)] and 2 objects whose names matched the names of the semantically related objects for word frequency. Yee and Sedivy (2006) also validated the items using a picture naming task to verify the pictures evoked the intended linguistic label and to ensure that the control items did not compete phonologically or semantically with other items on the display (see Yee and Sedivy, 2006, for a complete description of these pictures). The items were randomly placed in the grids in static positions a priori, which resulted in the same grid being viewed by each participant. However, the objects (target, semantic distractor, and their controls) were equally distributed in the grids across the experiment.

The 3-sentence discourses described the location of the objects in the grid and were played one after the other with a 600 ms delay between sentences starting after the display was shown for 2 s. Participants were given these 2 s in order to familiarize themselves with the objects and their location in the grid before hearing the auditory description, which they had to verify. Sentence 1 (~2454 ms) began by describing the location of the Target referent (one of the semantically related objects) in relation to an unrelated object (Sentence 1-mentioned) using definite references. Sentence 2 (~2294 ms) always referred to the Target using a pronoun and described the absolute position of the Target in the grid without referring to any other object. Sentence 3 varied by condition (*Pronoun* ~2025, *Repeated* ~2583 ms, *New* ~2595 ms), and was the critical sentence used for analysis.

Verbal stimuli were recorded by a native female speaker of American English (S.A.P.) and were edited using Adobe sound editing software. All the experimental items included the same recorded version of Sentences 1 and 2. Sentence 3 was recorded separately for each condition. Items were presented in a random order that was different for each participant. Each participant was presented with each experimental item once, such that they responded to eight items in each condition. Across all participants, each item appeared in each condition a similar number of times. Experimental items were always true, and 12 of the 48 fillers were also true, such that overall the verbal descriptions in exactly half of the trials were true. The false statement in the false filler items appeared exactly 12 times in each sentence position (1, 2, and 3).

### Apparatus

Participants' eye movements were recorded using a stationary chin rest ASL 6000 which sampled eye position at 240 Hz. Visual stimuli were presented on a 19” Dell CRT monitor positioned 62 cm directly in front of participants. The experiment was controlled by a Dell computer running the E-prime software (Schneider et al., 2002).

### Procedure

The experiment began with four practice trials, before which the experimenter calibrated the eye tracker using a 9-point calibration procedure. Calibration was repeated every four trials during the experiment if needed. This was done only when participants needed to exit the tracker momentarily for a break, or when visual analysis of drift indicated that participants had shifted head position. Participants were told that they would be looking at pictorial displays and hearing short discourses. They were instructed to listen to the discourses and decide whether they accurately described the displays. At the end of the recorded discourse, the pictorial display was replaced with a screen instructing participants to indicate their response by clicking on the words *True* or *False*. Participants were informed that even one false statement in the discourse made the entire discourse false. When participants clicked on a choice, they were given immediate feedback on their accuracy. Response accuracy was recorded and analyzed to ensure that participants were performing the task. The data from four participants whose accuracy was lower than 3 standard deviations below the median accuracy of all participants were removed from the analysis. In addition, 3 participants were also removed because proper calibration was not maintained throughout the course of the experiment. The data from the remaining 42 participants are reported below.

### Eye tracking data analysis

Raw eye position data from each participant were transformed into fixations using ASL Results 2.0 analysis software, following the procedure recommended by ASL. Fixations were then matched with the E-prime data file of the participant in order to determine the sentence, item, condition, and object fixated for each fixation. Proportions of fixations to each display item type were then calculated for each subject in each condition in consecutive 25 ms time windows by averaging across the experimental items the participant saw in each condition. These proportions of fixations to display items were the dependent variables for the analyses described below.

### GCAs

Because our focus was on the time course of processing the different reference form we chose to employ GCA of the fixation proportion data, following the procedure outlined in Mirman et al. (2008) and Mirman (2014). Unlike traditional analyses of variance, GCA includes time as a predictor in the model and analyzes the effect of the different conditions on the rate of change in fixation proportions. To ensure that our fixation proportion results were not biased by the relatively small number of observations per condition we repeated our analyses using the empirical logit transformations. As the results were identical and only served to increase power and fit, we only report the more readable proportion of fixations results.

Our analyses aimed to test the predictions of the different theories and focused on rapid changes in a short, 500 ms time window following the critical events in the sound files. For testing the lexical semantic competitor effect, in keeping with previous research (e.g., Yee and Sedivy, 2006), the window of analysis was 500 ms, starting 100 ms before the offset of the reference. This start point was maintained to capture the effect, as it has been shown to disappear rapidly when the competitor does not receive further mention. We also utilized a 500 ms time window for testing the consideration of possible referents after hearing the target reference; in order to better compare the effect of hearing pronouns, which are short, to the effect of hearing longer definite references, the window starting at the offset of the critical reference. Thus, the choice of this relatively short time window was based on our focus on the processes that occur immediately after listeners process the critical reference and minimize as much as possible the effect of inter-item differences in the subsequent linguistic input. While these analyses have been previously used in studies of single word processing (e.g., Chen and Mirman, 2015) and shifts in attention during conversation (Boiteau et al., 2014), they have not been used specifically in the type of reference processing comparison we present here. We chose to use these analyses as they allowed us to look closely at the rapid changes in processing and attention that are predicted by some of the contrasted hypotheses. While this short time window can only include one or two fixations, the high sampling rate and the averaging of items and subjects resulted in a rich data set that adequately reflected changes to the sustained attention to the different objects. Indeed, if these data were to be compared using a simple ANOVA, we would lose information regarding the finer differences in the processing of the different anaphoric expressions. An example of this type of finely tuned information is the average rate at which participants switch from looking from one display item to another. This rate of change can reveal the strength of the attentional commitment to a referent of an anaphoric expression just heard, which can be directly modeled as the quadratic component of a GCA. In contrast, this rate of change is not directly expressed in a traditional ANOVA, unless the means of consecutive *post-hoc* sized time windows are compared, with a likely loss of statistical power.

In our GCA, the models contained two levels, the first of which (Level-1, see top line in Equation 1), captures the effect of time on the performance of participant *i* in condition *j*:

(1) $$\begin{array}{l} Y_{ij}=\beta_{0i}+{\beta_{1i}}^{*}\;Time_{ij}+{\beta_{2i}}^{*}\;Time^{2ij}+{\beta_{3i}}^{*}\;Time^{3ij}+\varepsilon_{ij}\\ \;\beta_{0i}=\gamma_{00}+{\gamma_{01}}^{*}\text{ Condition}+\zeta_{0i}\\ \;\beta_{1i}=\gamma_{10}+{\gamma_{11}}^{*}\text{ Condition}+\zeta_{1i}\\ \;\beta_{2i}=\gamma_{20}+{\gamma_{21}}^{*}\text{ Condition}\\ \;\beta_{3i}=\gamma_{30}+{\gamma_{31}}^{*}\text{ Condition} \end{array}$$

In these models, the first order (linear/slope) effects of time (Time) reflect the overall rate of fixation change while second order (quadratic) effects (Time^2^) reflect the rise and fall of the change in fixation rate, and third order (cubic) effects (Time^3^) reflect higher order changes in the change rate of fixation rates (Mirman et al., 2008). Since we were interested in fixations on target objects over short time windows, which included non-linear change trends but not ones that were highly complex, all the models we tested included fixed effects of time up to the third power, as shown in Equation 1, although when less complex models were identified as having better fit, they were chosen.The second level in GCA is used to estimate the effect of condition on the intercept (γ_01_ in the second line of Equation 1) and on the time course at the different orders (γ_*k*1_ in the lines 3–5 in Equation 1) by adjusting for individuals and conditions. Our models always included a random effect of participants on the intercept (ζ_0*i*_ in line 2) and slope (ζ_1*i*_ in line 3), thus allowing both the estimated baseline fixation proportion and rate of change in fixations to vary across individuals, which serves to measure of the variability across participants within the model.

In GCA, the effect of the condition is inferred by its necessity for the fit of the model in a process of model comparison. The best-fitting model is chosen according to a criterion that optimizes model fit and number of degrees of freedom, such that the simplest model that fits the data no worse than more complex models is chosen. Here, again following Mirman et al. (2008), complexity of models varied by the order of the time coefficient included in the model. Within our analyses, the time variables were represented by orthogonal, mean centered polynomials in order to eliminate the possible confounding effects of multicollinearity. Note that due to the centering of the time variables, intercept coefficients represent the middle time point in the analyzed time range (e.g., 250 ms from the start of the time window) and not the first time point.

Despite its advantages in analyzing change, as in any analysis that is based on model comparison, the use of GCA carries a risk of over fitting the data. To reduce this risk, statistical texts recommend that the results of GCA are interpreted by considering the terms included in the chosen model, the parameter estimates within the chosen model, and the visual inspection of the fitted model (Long, 2012). Following this advice and in order to help readers interpret our results and analyses we provide the details of all three for each analysis. The selected models' parameter estimates are shown in tables within the paper. These tables also present *p*-values of individual coefficients, which we calculated following Mirman (2014), using a Unit Normal Table approximation to the critical *t*-values. Together with these tables we also provide figures with the best-fitting growth curve model overlaid on the mean observed. To facilitate the flow of presentation in the paper, the process of model selection including the differences in the fit of the contrasted models, are documented in the table section of the Supplemental Materials. Thus, the analyses in Supplemental Materials correspond with the tables included within the results, and offer the interested reader additional model fit information.

We should emphasize that our main goal is to compare the time course of reference processing through the sustained attention to referents in the different conditions. Therefore, our analyses focus on whether the best-fitting model includes any interaction effects involving condition and the intercept, or condition and any of the time terms.

Analyses were carried out using the R statistical software package (v.3.1.1, R Core Team, 2014), and the lme4 (Bates et al., 2014) and lmerTest (Kuznetsova et al., 2014) software packages, which run mixed-effects models including the GCAs used here. In order to avoid intractably complex analyses with high order interactions, we performed a series of planned analyses on subsets of the data aiming to test the specific theoretical predictions outlined above.

## Results

Eye tracking data from 42 participants were preprocessed and analyzed as described above. We present below the analyses of the fixations during Sentences 1 and 3.

### Sentence 1

Sentence 1 fixations were analyzed to replicate Yee and Sedivy's semantic competitor effect (2006). The current study utilized references to target objects in non-imperative, descriptive sentences. As Yee and Sedivy found that listeners looked at the Semantic Distractor after the offset of the target word, we carried out a GCA contrasting the fixations to the Semantic Distractor to fixations to Sentence 1-mentioned and to Sentence 3-mentioned during a 500 ms time window starting 100 ms before the offset of the Target referent. The participant had not yet heard the name of the three other items, and so any advantage for the Semantic Distractor (*mouse*) over the other two items (*bed* and *pump*) during this period would necessarily reflect a semantic competitor effect. As in all the analyses in this paper, all the contrasted models included level-1 terms corresponding to intercept, slope, and linear, quadratic, and cubic terms for Time. Here the model also included terms representing the destination of the fixation [Semantic Distractor (*mouse*), Sentence 1-mentioned (*bed*), or Sentence 3-mentioned (*pump*)], and their interaction with the various time terms.

**Figure 2:**
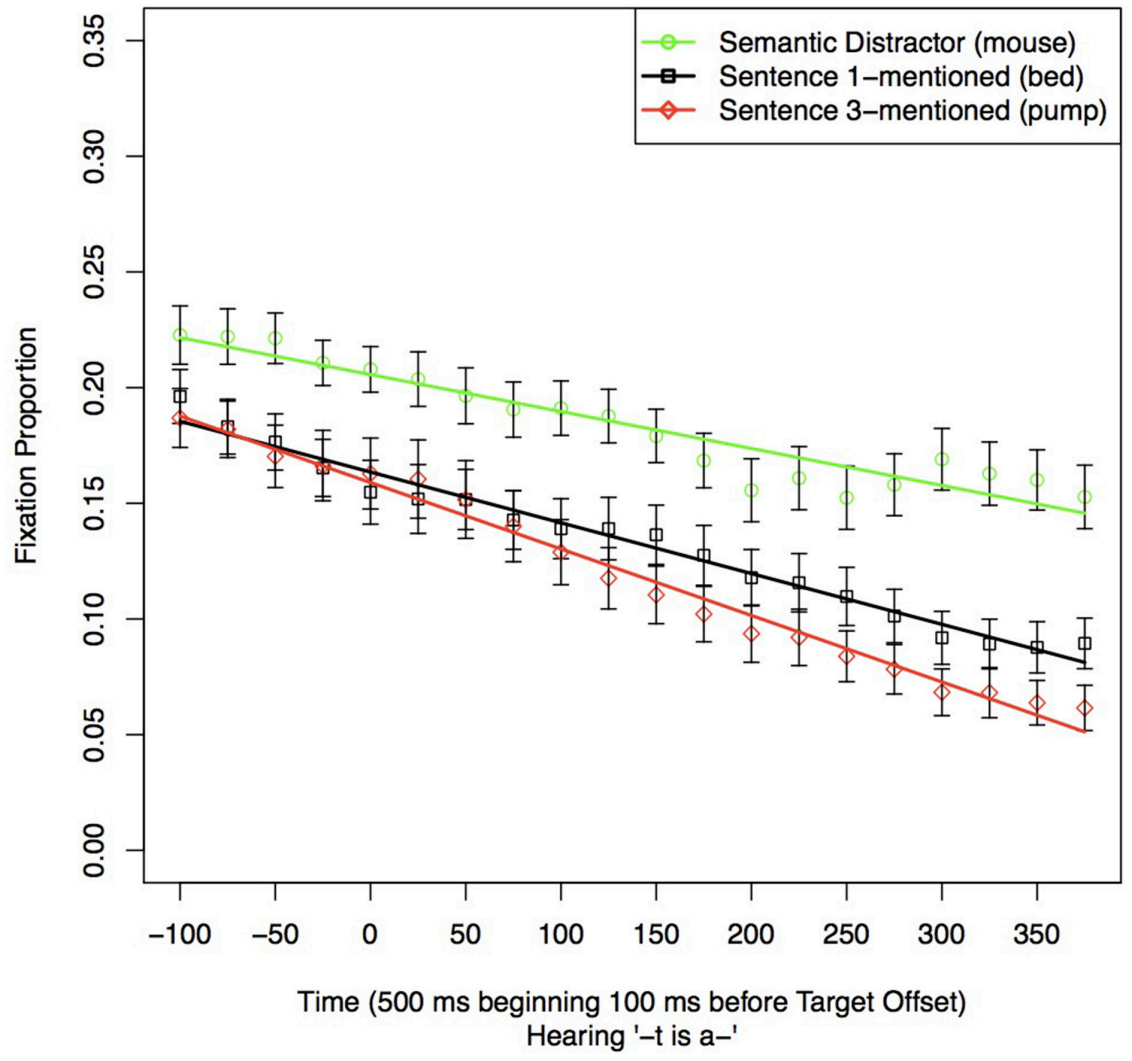
**Proportion of fixations to other items in the display in the 500 ms time window starting 100 ms before the offset of the reference to the Target in Sentence 1**. Error bars indicate the standard error of the mean for the condition across subjects in the time-window.

The coefficient estimates of the best-chosen model are given in Table 2 (see the correspondingly numbered tables in Supplemental Materials for the model comparison leading to the model's choice). Figure 2 shows the proportion of fixations to these three pictures as well as the fit estimate lines for the best-fitting model. For the remainder of our analyses, we will list these components in the same order. In the chosen model, the destination of fixation only affected the intercept and the linear time term, but not any of the higher order time terms.

**Table 2:** **Coefficient estimates in the best-fitting model, (slope) for proportion of fixations to the Semantic Distractor, Sentence 1-mentioned (S1-M), and Sentence 3-mentioned (S3-M) in Sentence 1 in a 500 ms time window starting 100 ms before the offset of the Target**.

**Coefficient**	***Est*.**	***Std. Error***	***t***	***p <***
Intercept	0.1837	0.0061	30.173	0.001
Time	−0.1030	0.0150	−6.884	0.001
S1-M	−0.0503	0.0034	−14.596	0.001
S3-M	−0.0642	0.0034	−18.624	0.001
Time^*^S1-M	−0.0385	0.0154	−2.494	0.01
Time^*^S3-M	−0.0822	0.0154	−5.334	0.001

*Proportion of fixations to the Semantic Distractor provided the baseline group*.

This analysis shows that, at the offset of the Target (*The cat*), listeners looked reliably more often and were slower to look away from the Semantic Distractor (*mouse*) than the other two objects that have not been mentioned yet (*bed* and *pump*). This analysis thus confirms that the semantic competitor effect observed by Yee and Sedivy (2006) occurs in declarative sentences like the ones used here.

### Sentence 3

#### Prediction 1

In order to test DPT's prediction that repeated references are processed like new references, we looked for differences between the Repeated and New conditions. Because these two conditions differed in whether the first reference in the critical third sentence was to the Target (Repeated condition, *The cat*…) or to Sentence 3-mentioned (New condition, *The pump*…), we only looked at fixations to pictures of the other two items: the Semantic Distractor (*mouse*), which was not mentioned in any of the conditions, and Sentence 1-mentioned (*bed*), which was mentioned in the first sentence in all the conditions. This ensured that any differences in fixations between the Repeated and New conditions do not merely reflect a baseline difference between looks to a picture that was mentioned before vs. one that was not. According to DPT, both the Repeated and New conditions should be similar and both should differ from the Pronoun condition.

Our first analysis contrasted the proportion of fixations to the Semantic Distractor (*mouse*) in the three conditions in the 500 ms time window following the offset of the critical first reference in Sentence 3. This time window was chosen because it captures mainly the effects of processing the first reference on eye movements and our focus in this analysis was on whether the initial and immediate processing of new and repeated references is similar. In all conditions, the Semantic Distractor has not been mentioned. If repeated and new references are processed similarly, then there should be no differences between the two conditions in fixations to an object that had still not been mentioned and both these conditions should differ from the Pronoun condition.

**Figure 3:**
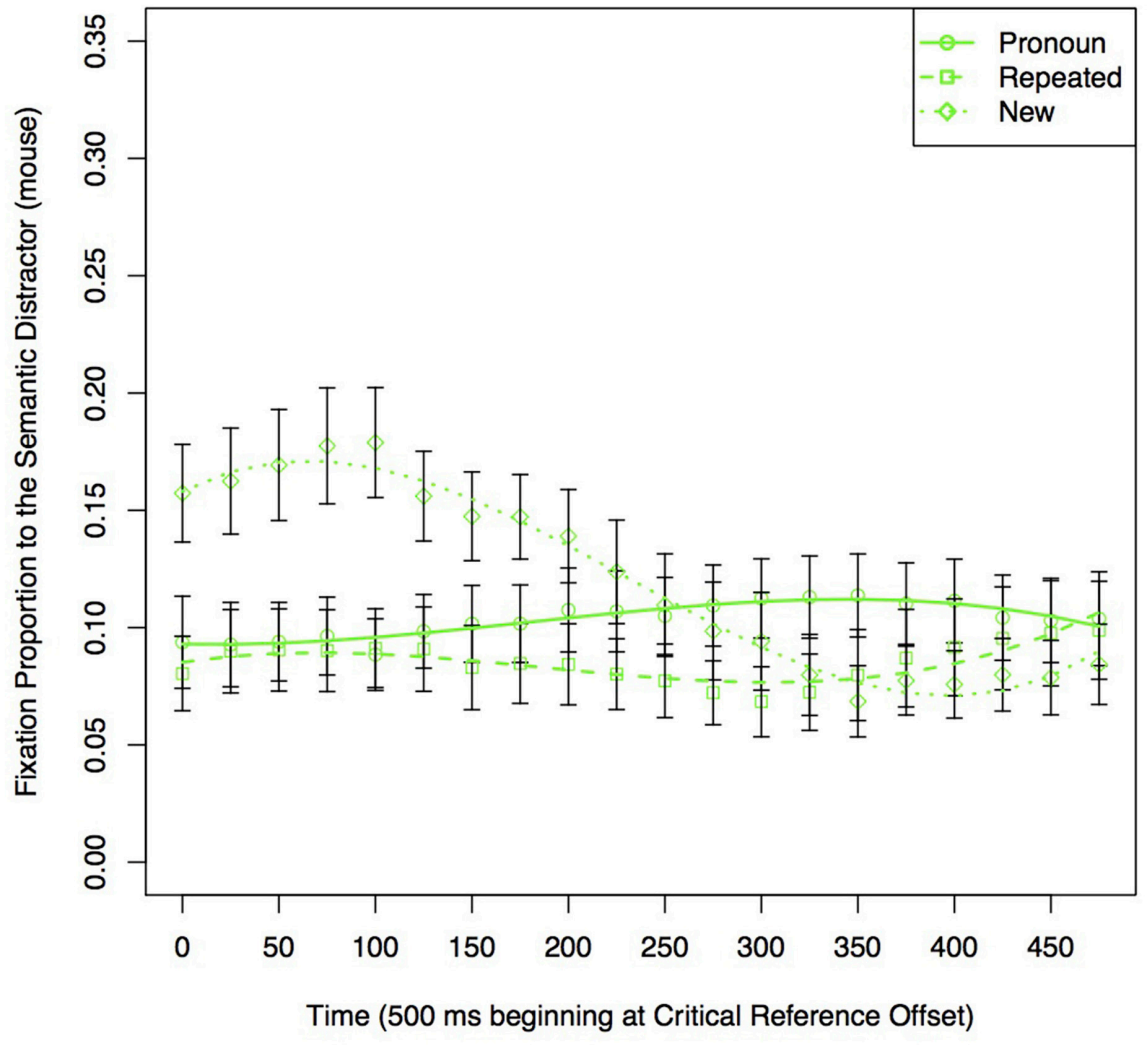
**Proportion of fixations to the Semantic Distractor picture (e.g., “the mouse”) in the display in Sentence 3 in the 500 ms time window after the offset of the critical reference (“the cat”)**. Fixations are graphed by condition: either Pronoun, Repeated, or New.

The results of the analyses are shown in Table 3A and Figure 3. As is shown, participants looked more at the Semantic Distractor in the New condition than in either the Repeated condition or the Pronoun condition in the first 250 ms, even though it was not mentioned in any of these conditions. The best-fitting model was cubic. This analysis illustrates the importance of using GCA, as averaging fixations over the time window would have likely missed this effect, and would not have provided any information about the dynamics of the fixations.

**Table 3:** **Coefficient estimates for the best-fitting models in the 500 ms time window starting at the offset of the critical reference in Sentence 3 to the (A) Semantic Distractor (cubic) and (B) Sentence 1-mentioned (slope) in the Pronoun, Repeated and New conditions**.

**Coefficient**	***Est*.**	***Std. Error***	***t***	***p <***
**(A) SEMANTIC DISTRACTOR**				
Intercept	0.0853	0.0088	9.744	0.001
Time	0.0045	0.0225	0.200	*n.s*.
Time^2^	0.0245	0.0159	1.542	*n.s*.
Time^3^	0.0205	0.0159	1.287	*n.s*.
Pronoun	0.0181	0.0050	3.600	0.001
New	0.0350	0.0050	6.963	0.001
Time^*^Pronoun	−0.0193	0.0225	0.860	*n.s*.
Time^*^New	−0.1645	0.0225	−7.317	0.001
Time^2^^*^Pronoun	−0.0399	0.0225	−1.773	0.08
Time^2^^*^New	−0.0158	0.0225	−0.705	*n.s*.
Time^3^^*^Pronoun	−0.0319	0.0225	−1.418	*n.s*.
Time^3^^*^New	0.0363	0.0225	1.616	0.11
**(B) SENTENCE 1-MENTIONED**				
Intercept	0.1352	0.0128	10.558	0.001
Time	0.0572	0.0262	2.182	0.05
Pronoun	−0.0373	0.0050	−7.380	0.001
New	−0.0621	0.0050	−12.297	0.001
Time^*^Pronoun	−0.0501	0.0226	−2.218	0.05
Time^*^New	−0.1581	0.0226	−7.000	0.001

*Proportion of fixations in the Repeated condition provided the baseline group*.

Our next GCA contrasted the proportion of fixations to Sentence 1-mentioned (*bed*) in the three conditions in the same time window. In all conditions, Sentence 1-mentioned was mentioned together with the Target (*cat*) in Sentence 1. If repeated and new references are processed similarly, then there should be no differences between the two conditions in fixations to an object that was previously mentioned.

**Figure 4:**
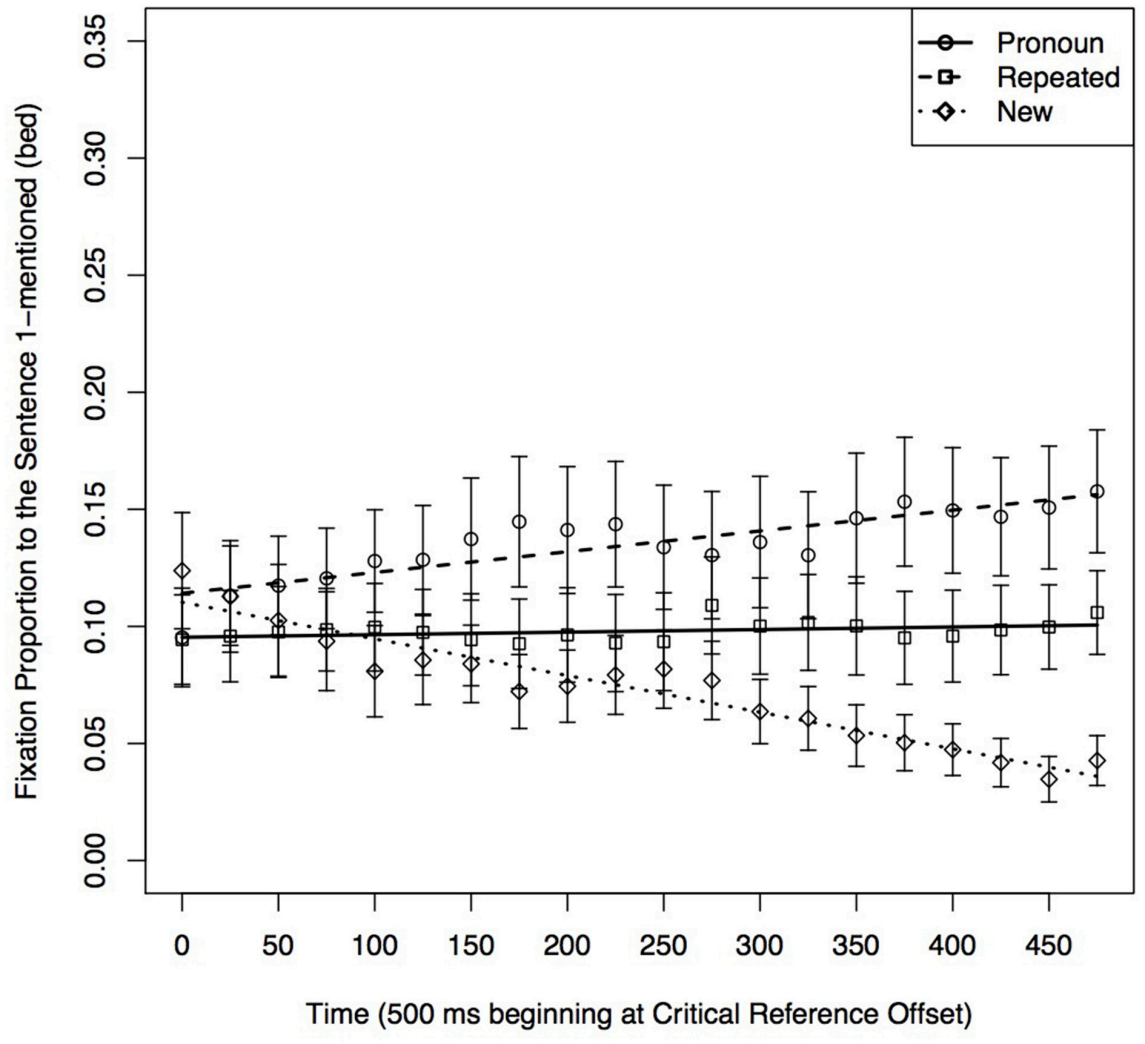
**Proportion of fixations to the Sentence 1-mentioned picture (bed) in the display in Sentence 3 in a 500 ms time window after the offset of the critical reference**. Fixations are graphed by condition, Pronoun, Repeated, or New.

The results of the analyses are shown in Table 3B and Figure 4. As is shown in both table and figure, there was an intercept effect reflecting more looks at Sentence 1-mentioned (*bed*) in the Repeated condition than in either the New or Pronoun conditions. There was also an effect of condition on slope reflecting a contrast between the steady increase in fixations to the Sentence 1-mentioned in the Repeated condition across the time window, in comparison to the steady decrease of looks in the New condition and the barely unchanged rate of looks in the Pronoun condition.

Together, the two analyses indicate that listeners process repeated and new references quite differently: they consider other referents from the previous sentence after hearing a repeated reference and consider new referents besides the one mentioned, when hearing a reference to a previously unmentioned referent. Thus, overall, in contrast to the predictions of DPT, repeated and new definite references were not processed similarly, in that repeated definite references increased fixations to a referent that was previously mentioned (Sentence 1-mentioned, *bed*), but new definite references did not.

#### Prediction 2

To test the predictions of the ILH that pronouns and repeated references are interpreted as referring to the target but repeated names lead to interference, our remaining analyses focused on differences between the Pronoun and Repeated conditions. The New condition was not included because the Target referent was not mentioned first. For clarity, we start with a separate analysis of fixations toward each of the 4 displayed objects in the Pronoun vs. Repeated conditions.

##### Target (cat)

**Figure 5:**
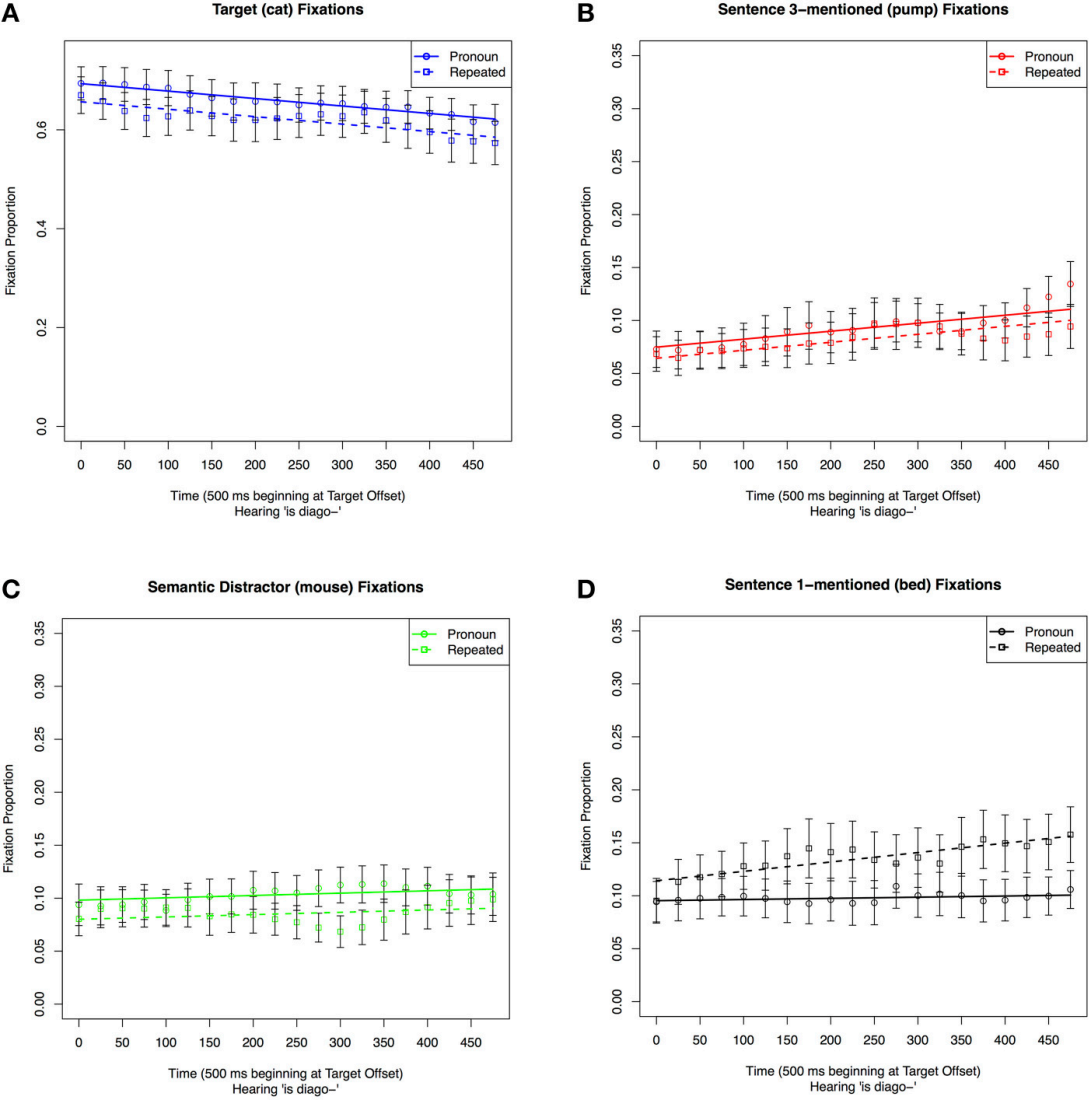
**Proportion of fixations to individual pictures on the display in the 500 ms time window starting at the offset of the reference to the Target in Sentence 3**. Fixations are graphed by anaphor form condition (Pronoun vs. Repeated): **(A)** Fixations to the pictures of the Target (cat); **(B)** Fixations to the picture of Sentence 3-mentioned (pump); **(C)** Fixations to pictures of the Semantic Distractor (mouse); **(D)** Proportion of fixations to the picture of Sentence 1-mentioned (bed).

First, in order to test whether looks to the target differed following pronoun and repeated references, we analyzed fixations to the Target (*cat*) in the Pronoun and Repeated conditions. The results of the analyses are shown in Table 4A and Figure 5A. As is shown in both table and figure, participants looked more often at the Target in the Pronoun condition than in the Repeated condition, but this tendency did not change across the 500 ms time window. Thus, while there were more looks to the Target in the Pronoun than in the Repeated condition, the rate of looking away from the Target as Sentence 3 unfolded was comparable in the two conditions.

**Table 4:** **Coefficient estimates in the best-fitting models of proportion of fixations to display items in the Pronoun and Repeated conditions in a 500 ms time window starting at the offset of the critical reference in Sentence 3: (A) the Target (cat; Model 1); (B) Sentence 3-mentioned (pump; Model 2); (C) Semantic Distractor (mouse; Model 3); (D) Sentence 1-mentioned (bed; Model 4)**.

**Coefficient**	***Est*.**	***Std. Error***	***t***	***p <***
**(A) MODEL 1. TARGET**				
Intercept	0.6576	0.0297	22.670	0.001
Time	−0.0971	0.0389	−2.498	0.05
Repeated	−0.0367	0.0075	−4.888	0.001
**(B) MODEL 2. SENTENCE 3-MENTIONED**				
Intercept	0.0927	0.0134	6.943	0.001
Time	0.0486	0.0229	2.120	0.05
Repeated	−0.0105	0.0043	−2.448	0.02
**(C) MODEL 3. SEMANTIC DISTRACTOR**				
Intercept	0.1034	0.0115	8.960	0.001
Time	0.00142	0.0217	0.654	*n.s*.
Repeated	−0.181	0.0040	−4.485	0.001
**(D) MODEL 4. SENTENCE 1-MENTIONED**				
Intercept	0.0980	0.0156	6.269	0.001
Time	0.0007	0.0285	0.249	*n.s*.
Repeated	0.0373	0.0049	7.566	0.001
Time^*^Repeated	0.0501	0.0220	2.274	0.05

*Proportion of fixations in the Pronoun condition provided the baseline group*.

##### Sentence 3-mentioned (pump)

Second, in order to test whether the two reference types led to differences during the processing of the remainder of Sentence 3, we analyzed fixations to the picture of the item that was mentioned second in this sentence (Sentence 3-mentioned, *pump*). The results of the analyses are shown in Table 4B and Figure 5B. The chosen model included an effect of condition on the intercept. As is shown in both the table and figure, the intercept effect was due to participants looking at the second-mentioned entity in the critical sentence more often in the Pronoun than in the Repeated condition.

Overall, the first two analyses show that (1) participants looked less often at the Target (*cat*) in the Repeated condition than in the Pronoun condition, and (2) participants looked more often at Sentence 3-mentioned (*pump*) in the Pronoun than the Repeated condition. Together, we interpret these effects as showing that pronouns were associated with quicker processing of the target as well as quicker processing of the second mentioned referent in the sentence. In other words, the pronoun condition showed less interference than the repeated condition.

##### Semantic distractor (mouse)

Next, we reanalyzed fixations to the Semantic Distractor without the New condition. The results of the analyses are shown in Table 4C and Figure 5C. The chosen model included an intercept effect of condition. The intercept effect indicated a greater number of fixations to the Semantic Distractor in the Pronoun than in the Repeated condition.

##### Sentence 1-mentioned (bed)

We also reanalyzed looks to Sentence 1-mentioned with only the Pronoun and Repeated conditions in the 500 ms time window. The results of the analyses are shown in Table 4D and Figure 5D. This model included effects of condition on the linear Time component indicating that participants looked more often at Sentence 1-mentioned in the Repeated condition than in the Pronoun condition throughout the 500 ms following the offset of the Target, and the difference increased toward the end of the time window. We interpret this finding as an indication of a greater activation of the previously mentioned referent in the Repeated condition than in the Pronoun condition. The fact that this effect increased over time indicates that this interference became more pronounced as processing progressed.

#### Prediction 3

The analysis above (Sentence 1-mentioned, *bed*) also tests Prediction 3. This prediction suggested that in the Pronoun and Repeated conditions, after hearing the Target, there should be fewer looks to the Sentence 1-mentioned than to other items that have not been mentioned. There are instead more looks to this item, particularly in the Pronoun condition.

## Discussion

Our data are not compatible with the DPT prediction of similar processing of new and repeated references. In our experiment, the New and Repeated conditions led to distinctively different fixation patterns to both the Semantic Distractor (*mouse*) and Sentence 1-mentioned (*bed*). The Semantic Distractor was not mentioned previously and, according to DPT, should have been considered a good “new” referent in both conditions. Sentence 1-mentioned was mentioned in the previous discourse, and, according to DPT, should not have been considered a good “new” referent in both conditions. Critically, this comparison did not involve looks to the Target which was a previously mentioned item in the Repeated condition and an unmentioned item in the New condition. Thus, there is no reason for concern that the differences we found reflect a difference between looks to an item that was mentioned before and one that was not.

We interpret the remainder of our findings as a manifestation of the RNP in that the Repeated condition led to delayed processing of information relative to the Pronoun condition. This was reflected in the smaller number of fixations to the second mentioned item in the critical sentence, and the increasingly greater number of fixations to the previously mentioned item in the Repeated condition than in the Pronoun condition. We note that, in line with the general claim of the ILH, this effect appeared related to an activated memory representation interfering with processing. However, this interference was associated with a competition driven by the activation of other previously mentioned referents and not, as the ILH had originally claimed, with the activation of broad semantic representations, as participants did not look more often to the Semantic Distractor in the Repeated condition relative to the Pronoun condition. In fact, participants looked *less* often at the Semantic Distractor initially in both conditions than in the New condition. A possible objection to this interpretation is that the new and repeated nouns related to different pictures such that when participants heard “cat” for the second time, they had likely looked previously at the picture of the cat and may also looked at the semantically related mouse. As a result, participants may have had less reason to identify and process the pictures of the cat and mouse again, and instead, they looked at the bed. We will return to discuss this objection in the context of the results of Experiment 2.

## Experiment 2

The results of Experiment 1 support the presence of the RNP in spoken language comprehension and the ILH's general claim about the involvement of memory interference related to the activation of other information. These results show that it is the activation of information that was associated with the referent in the previous discourse (Sentence 1-mentioned) that underlies the memory interference in the RNP. One way this finding could be explained is in terms of a cue-based theory of memory retrieval. Specifically, the mention of both referents in Sentence 1 may have created a representation of the two as a cue-retrieval target pair or at least combined some information about the Sentence 1-mentioned item with the discourse representation of the Target. Under a cue-based theory of memory retrieval, this may have resulted in the automatic retrieval of the Sentence 1-mentioned representation upon hearing the Target in Sentence 3, and this irrelevant retrieved information, caused the delay in processing. This retrieval process may have been more effective following repeated references because the extra information in these references may have provided a stronger retrieval cue.

Importantly, in contrast to the specific prediction of the ILH (Almor, 1999), Experiment 1 did not show any evidence of semantic effects in the Repeated condition. Thus, the results of Experiment 1 can be explained on the basis of a general memory mechanism, rather than the activation of pre-existing semantic relations in long-term memory. Given that previous research in reading has shown the involvement of long-term memory semantic relations in the RNP in reading (Almor, 1999; Cowles and Garnham, 2005), we wanted to further explore the absence of a similar effect here. Specifically, we wanted to ascertain whether a pre-existing semantic relation can modulate and interact with the retrieval and activation of the discourse representation of the referent in the future, perhaps causing interference when deciding upon a new referent, when it has been previously activated. For example, if *cat* and *mouse* are mentioned together, can the fact that the two have a pre-existing semantic relationship affect recall of *mouse* when *cat* is mentioned again later, vs. if *cat* and *bed* were originally mentioned together (as in Experiment 1). Experiment 2 therefore tested whether the processing of repeated reference is affected by the strength of the semantic relation between the referent and the information associated with it earlier in the discourse.

The design of Experiment 2 followed Experiment 1 closely except that it manipulated whether the other object mentioned with the Target in Sentence 1 was an unrelated object (the *Unrelated* condition), or the Semantic Distractor, (the *Related* condition). For clarity purposes, we will from now on refer to the unrelated object as *Sentence 1-unrelated*. Sentence 3 in Experiment 2 appeared only in two conditions: *Pronoun* and *Repeated*. The New condition from Experiment 1 was not of interest for the question at hand and therefore was not included. Table 1B shows a sample item. Experiment 2 used the same pictorial displays as in Experiment 1.

This experiment aimed to test the following specific predictions:

1. According to a simple cue-based retrieval explanation of reference processing, pre-existing semantic relations should facilitate the retrieval of the representation of the referent. Therefore, there should be more fixations to an item that is semantically related to the target referent (Semantic Distractor, *mouse*) than a comparable unrelated item (Sentence 1-unrelated, *bed*) when it was previously mentioned with the Target. This effect should be stronger for repeated names than for pronouns as repeated names provide a stronger retrieval cue.
2. According to the ILH, the Related and Unrelated Sentence 1 conditions should have a different effect on the processing of potential referents in the Repeated and Pronoun Sentence 3 conditions. Specifically, the interference in processing repeated anaphors is expected to be greater in the Related than in the Unrelated conditions, due to the increased activation of a related previously mentioned item (the Semantic Distractor in the Related conditions) than an unrelated previously mentioned item (the Sentence 1-unrelated in the Unrelated conditions). In the Repeated conditions, this should be reflected in more fixations that increase at a higher rate to the Semantic Distractor in the Related than in the Unrelated conditions. The ILH predicts that in the Pronoun conditions there will be no such differences.

As far as we can tell, DPT does not make any prediction about differences in processing between the Related and Unrelated conditions within either the Repeated condition or the Pronoun condition. Evidence of such differences is therefore unexpected according to DPT, but not necessarily incompatible with it.

Once again, our analyses involved GCAs. All data was preprocessed following the steps outlined in Experiment 1.

## Method

### Participants

Fifty-eight undergraduate students recruited from the University of South Carolina Psychology Department's participant pool participated in this experiment for course credit. All participants provided informed consent in accordance with the University's IRB. All participants were native speakers of American English.

### Materials

The pictorial displays used in Experiment 2 were identical to those used in Experiment 1 (see Figure 1). To help distinguish between the objects in this experiment, in which a different object was mentioned with the Target in Sentence 1, we refer to the picture labeled *Sentence 1-mentioned* in Experiment 1 as *Sentence 1-unrelated*. The 3-sentence discourses used for Experiment 2 were constructed by altering the experimental items used in Experiment 1. As in Experiment 1, the discourses described the location of the items and always left two items unmentioned until the final referent of Sentence 3 was identified. The first sentence of each discourse appeared in two conditions. Either an Unrelated condition (~2454 ms), in which, like in Experiment 1, the two referents were unrelated, or a Related condition (~2603 ms), in which the two referents were related. Indeed, the Unrelated condition simply used the first sentences from Experiment 1. In the Related condition, the second referent mentioned after the Target (*cat*) was the Semantic Distractor (*mouse*) instead of Sentence 1-unrelated (*bed*), and specified its location in relation to the Target. Sentence 2 was identical to Sentence 2 in Experiment 1. Sentence 3 contained only the Pronoun (~1957 ms) and Repeated conditions (~2563 ms), introducing a new referent (Sentence 3-mentioned, *pump*) as the second reference.

Verbal stimuli were recorded by the same native female speaker of American English (S.A.P.) and edited using sound editing software. All experimental items included the same version of Sentence 2. Sentences 1 and 3 were recorded separately for each condition. Items were presented in a random order, which differed by participant. Each participant heard each experimental item once such that they responded to six items in each condition. Across all participants, each item appeared in each condition a similar number of times. Experimental items were always true, and 12 of the 48 fillers were also true, such that overall the verbal descriptions in exactly half of the trials were true.

### Apparatus

The apparatus used in Experiment 2 was identical to that used in Experiment 1.

### Procedure

The procedure and task for Experiment 2 were identical to that of Experiment 1. Response accuracy for the task was again recorded; no participants were removed from analyses due to low accuracy within the task.

## Results

Raw eye position data transformation and condition matching were the same as in Experiment 1. Ten participants were removed before the analysis, due to equipment failure or poor calibration during the experiment, leaving 48 participants.

### Sentence 1

**Figure 6:**
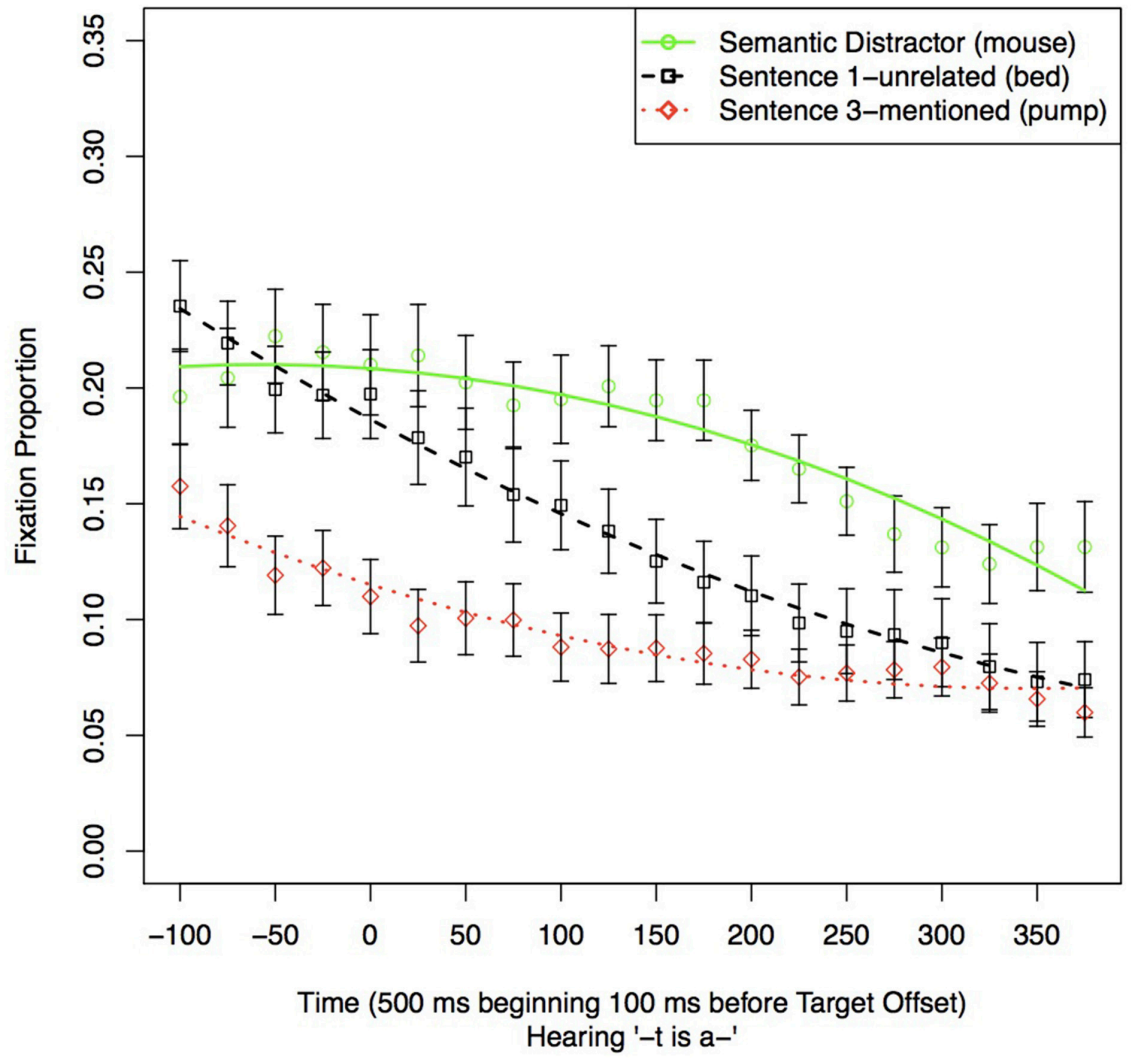
**Proportion of fixations to other items in the display in a 500 ms time window starting 100 ms before the offset of the Target reference in Sentence 1**. This figure replicates the semantic competitor effect observed in Experiment 1.

We tested for a replication of the semantic competitor effect from Experiment 1. We examined fixations during Sentence 1 in the Unrelated condition in the same time window as in Experiment 1. We only included the Unrelated condition because immediately at the offset of the Target, participants already began hearing the location of the second mentioned item, which in the Related condition was the Semantic Distractor. This made it impossible to gauge the effect of semantic relatedness in the Related condition. The results of the analyses are shown in Table 5 and Figure 6. The best-fitting model included effects of condition on both the linear and quadratic time terms. Coefficient estimates were close to those obtained in Experiment 1, with the exception that the effect of the Sentence 3-mentioned on the linear component of Time, which reversed in sign. The quadratic components indicate that the changes in proportion of fixation is different for fixations to the items. Combined, these data indicate a semantic competitor effect similar to the one observed in Experiment 1. The semantic competitor (Semantic Distractor) received more fixations than the other two objects not yet mentioned. The slight difference in results is not unexpected as the Semantic Distractor was never mentioned in Experiment 1, yet here, although not in the trials used to test for the effect, it was mentioned.

**Table 5:** **Coefficient estimates in the best-fitting quadratic model**.

**Coefficient**	***Est*.**	***Std. Error***	***t***	***p <***
Intercept	0.1794	0.0080	22.341	0.001
Time	−0.1313	0.0207	−6.332	0.001
Time^2^	0.0432	0.0153	−2.825	0.01
S1-U	−0.0397	0.0048	−8.225	0.001
S3-M	−0.0851	0.0048	−17.615	0.001
Time^*^S1-U	−0.0910	0.0216	−4.210	0.001
Time^*^S3-M	0.0377	0.0216	1.424	*n.s*.
Time^2^^*^S1-U	0.0727	0.0216	3.366	0.001
Time^2^^*^S3-M	0.0738	0.0216	3.414	0.001

*Comparisons for proportion of fixations to the Semantic Distractor, Sentence 1-unrelated (S1-U), and Sentence 3-mentioned (S3-M) in Sentence 1 in a 500 ms time window starting 100 ms before the offset of the Target in Sentence 1. Proportion of fixations to the Semantic Distractor provided the baseline group*.

### Sentence 3

#### Prediction 1

To test the cue-based retrieval explanation, we carried out analyses comparing fixations to an item previously mentioned with the Target when it was related to the Target (Semantic Distractor, *mouse*) to when it was not (Sentence 1-unrelated, *bed*). We did this separately for the Repeated and Pronoun conditions.

**Figure 7:**
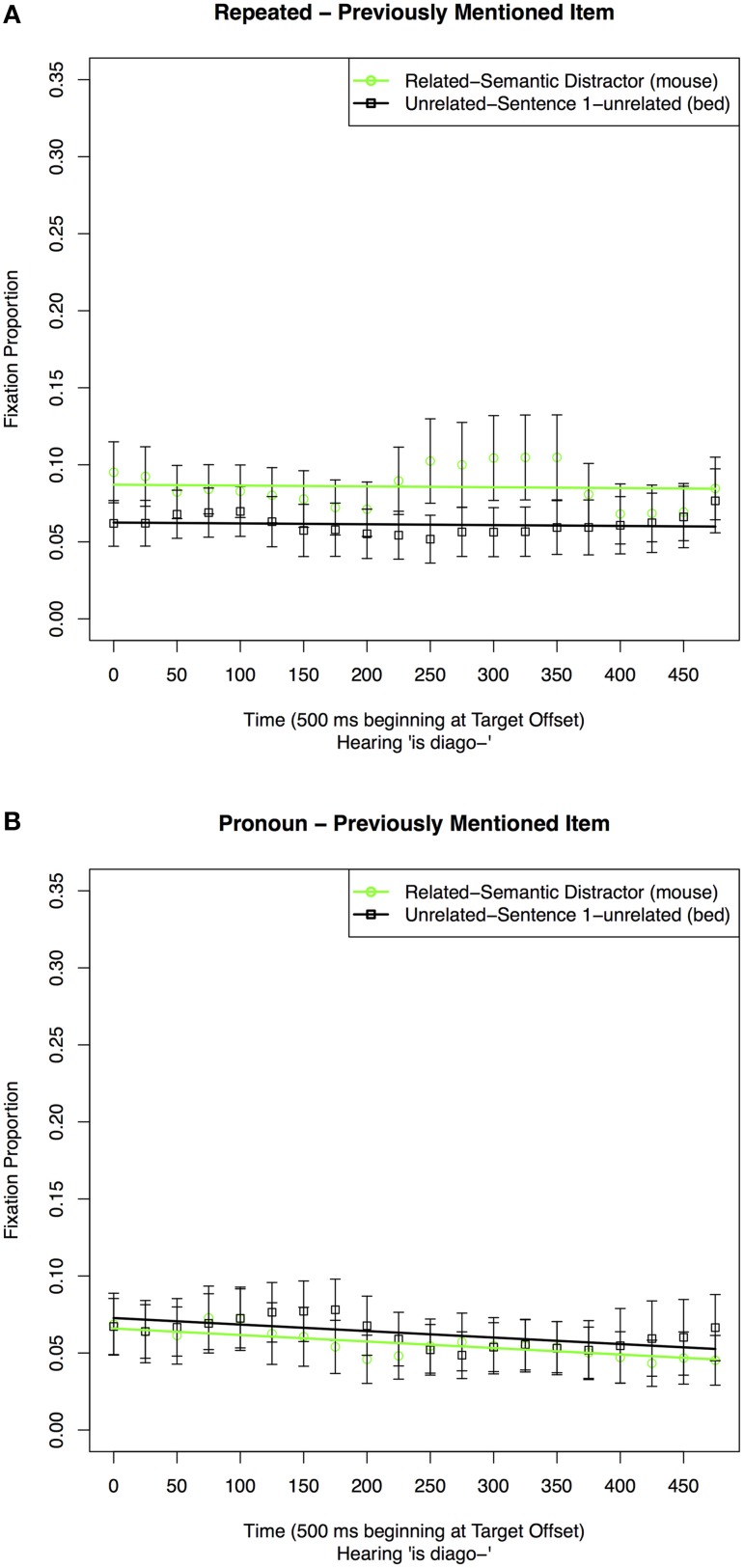
**Proportion of fixations to the items in the display previously mentioned with the Target in a 500 ms time window starting at the offset of the Target reference in Sentence 3 in the (A) Repeated and (B) Pronoun conditions**.

The results of the analyses are shown in Table 6 and Figure 7. For the Repeated conditions, when mentioned together with the Target in Sentence 1, there were more looks to an item that was semantically related to the Target (Semantic Distractor, *mouse*), than to an item that was not (Sentence 1-unrelated, *bed*) (Table 6, Figure 7A). This shows that a pre-existing semantic relation can modulate the interference caused by items mentioned earlier in the discourse. For the Pronoun conditions (Figure 7B) there were no differences in looks to the items dependent on Sentence 1 condition, so while the graph is included for illustrative purposes, a corresponding model does not appear. Thus, there is no evidence for interference caused by a pre-existing semantic relationship on pronoun resolution.

**Table 6:** **Coefficient estimates in the best-fitting model for proportions of fixations to items previously mentioned with the Target in Sentence 1 during Sentence 3 in the 500 ms time window following Target offset in the Repeated condition**.

**Coefficient**	***Est*.**	***Std. Error***	***t***	***p <***
Intercept	0.0858	0.0117	7.308	0.001
Time	−0.0035	0.0196	−0.189	*n.s*.
Unrelated-S1-U	−0.0246	0.0047	−5.184	0.001

*Models in the Pronoun condition demonstrated no significant difference. Proportion of fixations at the offset of the Target in the Related condition to the Semantic Distractor provided the baseline group*.

#### Prediction 2

We carried out analyses comparing fixations to each display item in the Related and Unrelated conditions of Sentence 1, first in the Repeated conditions and then in the Pronoun conditions. Because our focus in this experiment was on the effect of semantic relatedness, we chose to conduct a separate set of analyses for each type of referential expression. This approach was not used in Experiment 1, in which the informative comparisons were between different reference types.

##### Repeated condition: fixations to individual objects in the related vs. unrelated condition

##### Target (cat)

**Figure 8:**
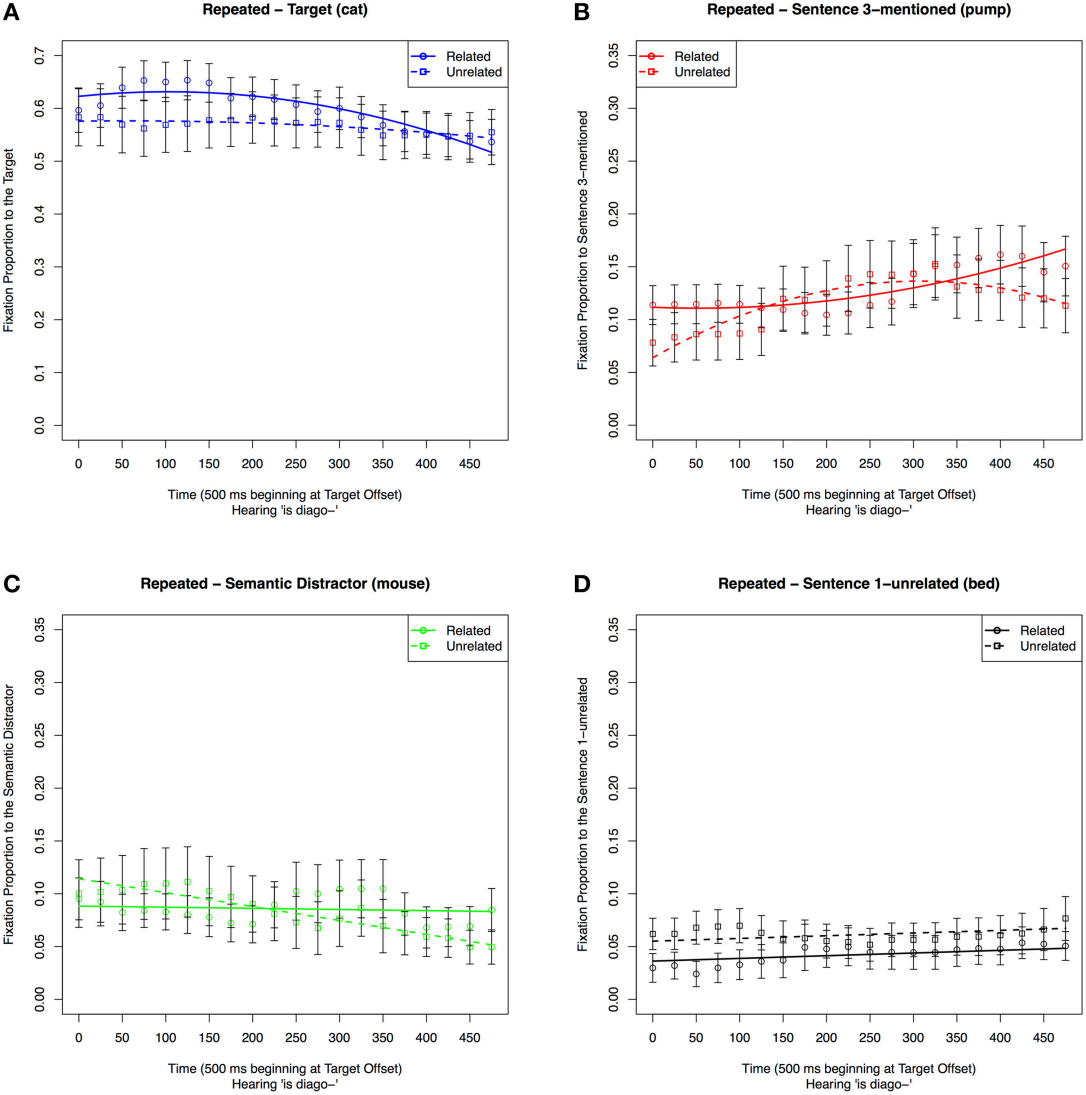
**Proportion of fixations in the 500 ms time window following Target offset to the (A) Target, (B) Sentence 3-mentioned, (C) Semantic Distractor, and (D) Sentence 1-unrelated in the Repeated conditions when the Sentence 1 condition was Unrelated or Related**.

The results of the analyses are shown in Table 7 Model 1A and Figure 8A. The chosen quadratic model included significant effects of condition on the intercept, slope and quadratic Time terms. These reflect participants initially fixating more on the Target and later fixating away from it sooner in the Related condition than in the Unrelated condition.

**Table 7:** **Coefficient estimates for the best-fitting models in the 500 ms time window following Target offset in Sentence 3 of a Repeated (Model 1) or Pronoun (Model 2) reference when Sentence 1 was in the Unrelated vs. Related condition, and proportion of fixations to the (A) Target, (B) Sentence 3-mentioned, (C) Semantic Distractor, and (D, Repeated only) Sentence 1-unrelated served as the outcome**.

**Coefficient**	***Est*.**	***Std. Error***	***t***	***p <***
**Model 1. Repeated**				
**(A) TARGET**				
Intercept	0.5993	0.0363	16.528	0.001
Time	−0.1438	0.0428	−3.360	0.01
Time^2^	−0.0685	0.0237	−2.897	0.01
Unrelated	−0.0328	0.0075	−4.391	0.001
Time^*^Unrelated	0.1006	0.0335	3.007	0.01
Time^2^^*^Unrelated	0.0533	0.0335	1.593	*n.s*.
**(B) SENTENCE 3-MENTIONED**				
Intercept	0.1280	0.0172	7.464	0.001
Time	0.0749	0.0351	2.134	0.05
Time^2^	0.0260	0.0186	1.395	*n.s*.
Unrelated	−0.0112	0.0059	−1.911	0.06
Time^*^Unrelated	−0.0054	0.0263	−0.207	*n.s*.
Time^2^^*^Unrelated	−0.0893	0.0263	−3.395	0.001
**(C) SEMANTIC DISTRACTOR**				
Intercept	0.0858	0.0171	5.022	0.001
Time	−0.0070	0.0344	−0.203	*n.s*.
Unrelated	−0.0029	0.0051	−0.568	*n.s*.
Time^*^Unrelated	−0.0778	0.0226	−3.438	0.001
**(D) SENTENCE 1-UNRELATED**				
Intercept	0.0423	0.0096	4.406	0.001
Time	0.0164	0.0127	1.291	*n.s*.
Unrelated	0.0189	0.0042	4.548	0.001
**Model 2. Pronoun**				
**(A) TARGET**				
Intercept	0.6135	0.0401	18.060	0.001
Time	−0.0119	0.0489	−0.314	*n.s*.
Unrelated	0.0182	0.0007	−6.238	0.001
**(B) SENTENCE 3-MENTIONED**				
Intercept	0.1262	0.0154	8.211	0.001
Time	0.0602	0.0322	1.870	0.07
Time^2^	0.0212	0.0179	1.181	*n.s*.
Unrelated	−0.0424	0.0057	−7.487	0.001
Time^*^Unrelated	−0.0700	0.0253	−2.762	0.01
Time^2^^*^Unrelated	−0.0177	0.0253	0.698	*n.s*.
**(C) SEMANTIC DISTRACTOR**				
Intercept	0.0559	0.0118	4.743	0.001
Time	−0.0322	0.0190	−1.693	*n.s*.
Unrelated	0.0216	0.0043	5.000	0.001
Time^*^Unrelated	0.0535	0.0193	2.764	0.01

*The Related condition served as the baseline*.

##### Sentence 3-mentioned (pump)

The results of the analyses are shown in Table 7 Model 1B and Figure 8B. Overall there were marginally more fixations to the Sentence 3-mentioned item in the Related condition. However, as shown in the graph and indicated by the quadratic effects of condition on Time, fixations in the Related condition rose over time, while fixations in the Unrelated condition rose and then fell in the same window.

##### Semantic distractor (mouse)

The results of the analyses are shown in Table 7 Model 1C and Figure 8C. The best-fitting model included a condition effect on the intercept and the slope Time term. While fixations to the Semantic Distractor increased with time in the Related condition, they decreased in the Unrelated condition.

##### Sentence 1-unrelated (bed)

The results of the analyses are shown in Table 7 Model 1D and Figure 8D. The best-fitting intercept only GCA model for these data included an effect of condition. There were more fixations to Sentence 1-unrelated in the Unrelated condition than in the Related condition.

##### Pronoun condition: fixations to individual objects in the related vs. unrelated conditions

##### Target (cat)

**Figure 9:**
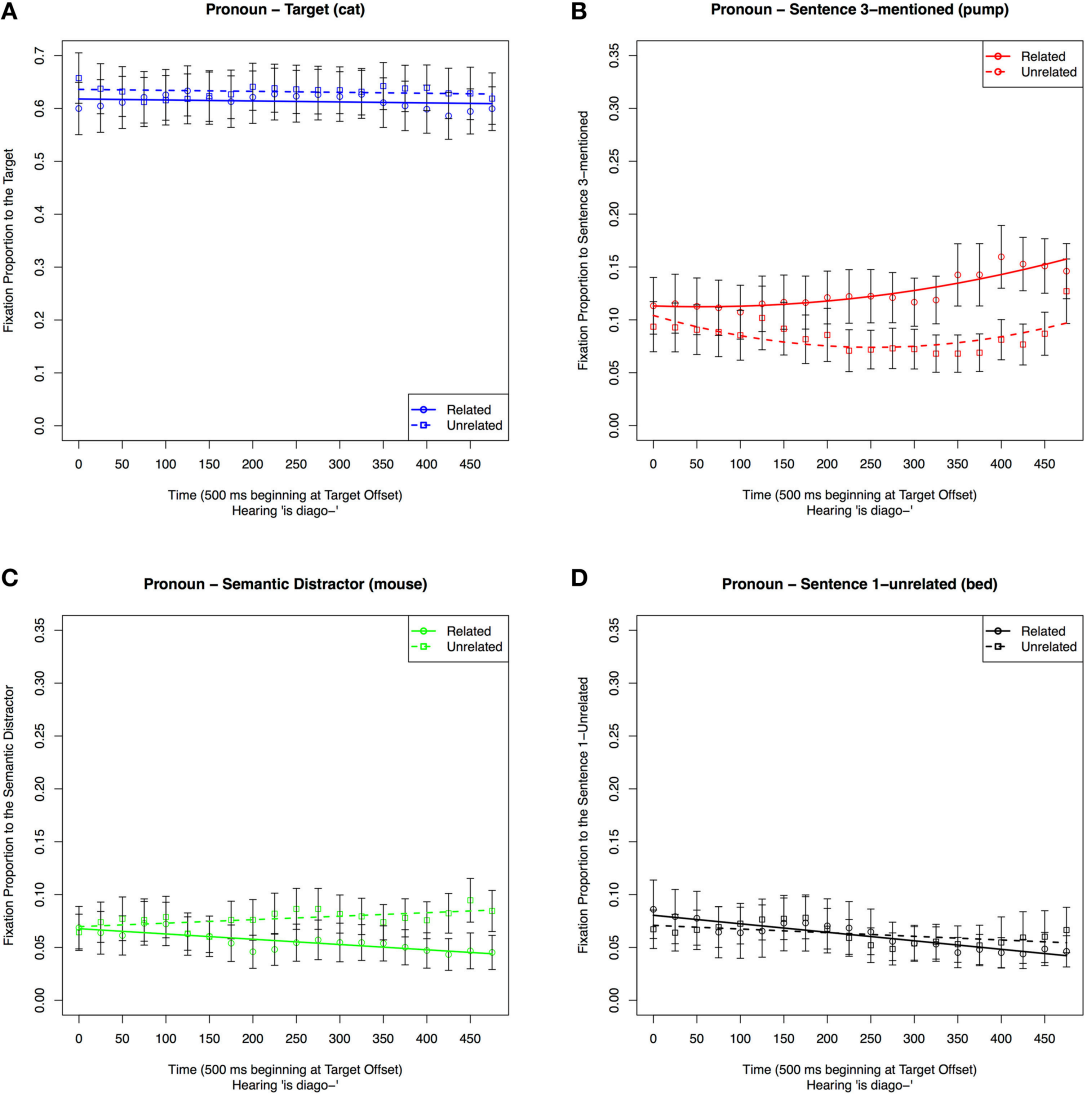
**Proportion of fixations to the (A) Target, (B) Sentence 3-mentioned, (C) Semantic Distractor, and (D) Sentence 1-unrelated in the 500 ms time window following Target offset in the Pronoun conditions when the Sentence 1 condition was Unrelated or Related**.

The results of the analyses are shown in Table 7 Model 2A and Figure 9A. The selected model included an effect of condition only on the intercept. Thus, as is shown in both table and figure, participants looked more often at the Target in the Unrelated condition than in the Related condition at the Target offset, but there were no differences in the time course of processing. This differs from the Repeated condition, where participants initially fixate on the Target in the Related condition, then fixate away from it at a quicker rate than the Unrelated condition.

##### Sentence 3-mentioned (pump)

The results of the analyses are shown in Table 7 Model 2B and Figure 9B. The selected quadratic model included an effect of condition on the slope Time coefficient. As is shown in both table and figure, participants looked more often and at an increased rate at Sentence 3-mentioned in the Related condition than in the Unrelated condition. This differed from the Repeated condition in which the Unrelated condition had an increasing and then decreasing pattern of fixations within the same time window.

##### Semantic distractor (mouse)

The results of the analyses are shown in Table 7 Model 2C and Figure 9C. The best-fitting model only included a significant effect of condition on the slope of the time parameter, with a quicker rate of fixating away from the Semantic Distractor in the Unrelated than in the Related condition. This differed from the Repeated condition, in which fixations in the Unrelated condition were lower and decreased over time.

##### Sentence 1-unrelated (bed)

The graphical result of the analysis is shown in Figure 9D. While the intercept model was graphed for full comparison purposes as it was the best fit, the model was not significant and is not included. Thus, there were not any differences between looks to Sentence 1-unrelated in the Related vs. Unrelated conditions following pronouns. This differs from the Repeated condition in which the item received more fixations in the Unrelated condition.

Overall these results show that when an item is initially mentioned with the Target, there are differences due to the semantic relation between that item and the Target on the later processing of a reference to the Target. In the case of a repeated reference, previously related items (*mouse*) receive more fixations than unrelated ones (*bed*). Also, for repeated references, mentioning an item related to the Target initially hinders performance and then has a facilitative effect. This could reflect the related item being considered as the next possible referent. In contrast, for pronouns, mentioning an item related to the Target facilitates resolution in comparison to mentioning an unrelated item. This could reflect the quicker dismissal of a related item than an unrelated item as a candidate for being the next possible referent.

## Discussion

The results of this experiment show clearly that pre-existing semantic relations between a referent and a previously mentioned item generally facilitate reference resolution in our paradigm. Following both pronouns and repeated definite references, a semantic relation between the target referent and a previously mentioned item facilitated processing. This was reflected in the higher rate of fixations to the referent mentioned next in Sentence 3 (Sentence 3-mentioned; *pump*) in the Related than in the Unrelated conditions at the end of the time window. However, despite the similarity in the effect of semantic relatedness at the end of the time window following pronouns and repeated definite references, there were important differences in the time course of this effect for the two reference types. While semantic relatedness consistently facilitated processing across the entire time window following pronouns, its effect on processing varied following repeated definite references. In particular, following repeated references, the higher fixation rate to Sentence 3-mentioned (*pump*) in the Related compared to the Unrelated condition occurred only in the last part of the time window.

These results support the predictions of the cue-based retrieval view (*Prediction 1*) in that following repeated names, but not pronouns, there were more fixations to the Semantic Distractor than to Sentence 1-unrelated when each was mentioned with the Target in Sentence 1 (Figure 7). The results are compatible with the ILH (*Prediction 2*) in that, following repeated names, there were more fixations that decreased at a slower rate to the Semantic Distractor in the Related than in the Unrelated condition. Also in line with this prediction, this pattern reversed following pronouns in that there were fewer fixations that decreased at a higher rate to the Semantic Distractor in the Related than in the Unrelated condition. It thus appears that for pronouns, semantic relatedness of a previously mentioned item resulted in the quicker rejection of inappropriate referents. For repeated names, the process was a bit more complex. When a previously mentioned item was semantically related to the referent, it was briefly considered a possible referent of the repeated reference, but was quickly discarded.

An alternative explanation for why participants often looked at the Semantic Distractor when they heard the Target may be due to automatic spreading activation between related concepts/words. In other words, participants may have suppressed “the mouse” as a potential antecedent for the “cat,” but may have nevertheless looked at the picture of “mouse” regardless of whether it could be a potential antecedent. Because pronouns are semantically related to neither the Target nor the Semantic Distractor, this did not happen after pronouns. While this interpretation provides a possible explanation for the results of Experiment 2, it is incompatible with the results of Experiment 1 in which the semantic distractor received more fixations in the Pronoun than in the Repeated condition. The results of Experiment 1 thus indicate that the effect in Experiment 2 is clearly related to the fact that the Semantic Distractor was mentioned in the discourse.

It should be noted that the activation of the previously unmentioned referents during the processing of repeated references is also compatible with the main tenant of DPT, which is that repeated reference is initially interpreted as a new reference. However, the finding that these activations are sensitive to the semantic relations between previously mentioned referents, is not predicted by DPT.

In the discussion of Experiment 1, we described an alternative explanation for the increased looks to the previously unmentioned item in the Repeated condition relative to the New condition. According to this alternative explanation, this difference merely reflected the greater likelihood that the Target and the Semantic Distractor were already looked at in comparison to the unmentioned referent. This explanation is incompatible with the finding in the current experiment that semantic relatedness increased this effect rather than weakened it as this alternative explanation would predict (given that participants were more likely to have previously looked at the Semantic Distractor than at Sentence-1-Unrelated).

## General discussion

Overall, our results indicate that an effect similar to the RNP observed in self-paced reading also occurs in spoken language comprehension. Our results also allow us to understand the time course and possible memory basis of this effect better than in previous reading studies. In the current study, this effect was reflected in delayed fixations to the second referent mentioned in the critical sentence following a repeated reference relative to a pronoun. Use of the VWP in conjunction with GCA techniques allowed us to examine the fine time course of the underlying processes, and demonstrate that the RNP is associated with discourse integration, which is delayed beyond the initial processing of the reference. Our results further show that such delays are related to the memory activation of discourse representations, and that this activation is influenced by a combination of previous mentions, semantic relations, and reference type. To our knowledge, our study is the first to use GCA analyses to better understand the time course of discourse reference in spoken language comprehension. We believe we have shown that using this type of analysis can be profitable for the understanding of these processes.

Our results provide mixed evidence regarding DPT (Gordon and Hendrick, 1998). In contrast to DPT's core claim that repeated references are processed like new references, Experiment 1 revealed that the two kinds of reference are processed differently. In that experiment, repeated references increased fixations to previously mentioned items, but new references increased fixations to items that were not previously mentioned. Nevertheless, the results of Experiment 2 provided some support for DPT in finding that previously unmentioned items were considered possible referents for a repeated reference. However, DPT does not predict the finding that this consideration was influenced by the semantic relation between the unmentioned items and the target referent. While this finding is not plainly incompatible with DPT, it does place this theory at a disadvantage relative to theories that do specifically predict semantic effects. Overall, while DPT's claim that repeated and new references are processed alike may be too simplistic, a weaker version of this claim may be true. The processing of repeated references may generally involve the consideration of previously unmentioned items, but mentioned items are considered first, and semantic representations play a role.

Our results support the general claim of the ILH (Almor, 1999, 2000, 2004; Almor and Nair, 2007) that the RNP is related to memory interference that delays the integrative processing of the reference. At the same time, the results also help clarify the nature of this memory interference. Specifically, our results show that this interference reflects the activation of prior information associated with the referent at the expense of ongoing discourse integration. Experiment 2 further showed that semantic relations play a role in this interference. When the two items that were mentioned together in Sentence 1 were semantically related, a pronoun reference was processed quicker and a repeated reference was processed slower. This suggests that processing both pronouns and repeated references involves activation of semantic discourse representations, although this activation affects the two reference types differently.

These findings can be explained in a cue-based memory framework. When two items are mentioned together, their discourse representations are more strongly connected when they are semantically related than when they are not. Therefore, a later mention of one of the items causes a quicker and stronger activation of the other when the two are related. This appears to have a different effect on the processing of pronouns and repeated names. Although it is possible that this is related to the consideration of the reasons for why, in the repeated condition, a repeated name has been used rather than a pronoun, this does not explain the specific patterns of results or provide any additional information about the underlying memory mechanism. Instead, we hypothesize that processing pronouns involves picking the most salient referent while actively suppressing other possible referents. The quicker activation of the representation of the other item in the related case allows for its quicker suppression as well, relative to the unrelated case. In contrast, processing repeated references involves a competition between the activated possible referents. Therefore, the stronger activation of a mentioned item when it is related to the Target relative to when it is not, leads to greater competition, causing a delay in processing. This explanation is compatible with the general claim of the ILH that the RNP reflects memory interference between semantic representations. However, unlike in previous work on the ILH, the interference here is caused by considering alternative and upcoming referents rather than by direct memory interference between the representations of the referent and the current reference.

The difference between the interference found in this study and the interference claimed by the ILH could be attributed to several factors. The first is the type of manipulation used in the present study vs. previous studies of semantic effects on reference processing. In contrast to the present study, several previous studies manipulated the semantic distance between a referential expression and the original mention of the referent (e.g., Sanford and Garrod, 1981; Garnham et al., 1997; Almor, 1999; van Gompel et al., 2004; Cowles and Garnham, 2005). Moreover, these studies focused on a hierarchical semantic overlap between the reference and the previous mention (e.g., *robin-bird* or *bird-animal*), whereas the semantic relations we examined here were based on a broader notion of semantic relatedness that did not involve hierarchical relations (e.g., *hammer-nail*). Thus, the interference found in the present study does not preclude the existence of other forms of interference, such as between semantically overlapping representations of referents and references.

In addition to the importance of these results for the two theories we tested here, we believe that our findings about the memory processes and activations associated with the different types of reference are novel and provide a meaningful empirical contribution to the literature. Overall, we have shown that reference processing reflects underlying memory representations and processes that, in line with general theories of memory, are affected by semantic relations and previous mention. A closer semantic relation between a previously mentioned item and a co-mentioned referent results in a stronger activation of the co-mentioned referent when a subsequent reference is encountered. For pronominal references, this stronger activation allows quicker suppression of the co-mentioned referent and therefore a quicker identification of the correct referent. In the case of repeated references, this stronger activation results in increased competition, which interferes with identifying the correct referent. Therefore, although pronouns and repeated references are processed differently, these differences can still be captured by general memory principles such as interference, suppression and competition. Finally, we believe that our novel use of GCA to study the processing time course of referential expressions provides a methodological contribution to the literature.

## Author contributions

All authors listed, have made substantial, direct, and intellectual contribution to the work, and approved it for publication.

### Conflict of interest statement

The authors declare that the research was conducted in the absence of any commercial or financial relationships that could be construed as a potential conflict of interest.
