# Supplementary material for: Semantic Relations Cause Interference in Spoken Language Comprehension When Using Repeated Definite References, Not Pronouns
Source: Front Psychol. 2016 Mar 1;7:214. doi: 10.3389/fpsyg.2016.00214 (PMC4772389; doi:10.3389/fpsyg.2016.00214)
Supplement: Supplementary file 1 [file DataSheet1.DOCX]

**Supplemental Table Information**

Table 2.

Model comparisons for models fit to the proportion of fixations to the Semantic Distractor, Sentence 1-mentioned, and Sentence 3-mentioned in Sentence 1 in a 500 ms time window starting 100 ms before the offset of the Target. Estimates for the base model (no conditions) are also given for comparison. The best fitting model is printed in bold.

Model Model fit

*AIC BIC logLik p* <

Base -5413.1 -5384.2 2711.6 -

Intercept -5818.5 -5772.2 2917.2 .001

**Slope -5842.8 -5784.9 2931.4 .001**

Quadratic -5841.8 -5766.6 2933.9 *n.s.*

Table 3.

Model comparisons for models fit to the proportion of fixations to the a) Semantic Distractor and b) Sentence 1-mentioned in the Pronoun, Repeated and New conditions in a 500 ms time window starting at the offset of the critical reference in Sentence 3. Estimates for the base model (no conditions) are also given for comparison. The best fitting model is printed in bold.

*a) Semantic Distractor*

Model Model fit

*AIC BIC logLik p* <

Base -3892.1 -3863.2 1951.0 -

Intercept -3937.5 -3891.2 1976.7 .001

Slope -4012.2 -3954.4 2016.1 .001

Quadratic -4009.8 -3934.6 2017.9 *n.s.*

**Cubic -4018.7 -3926.2 2025.4 .002**

*b) Sentence 1-mentioned*

Model Model fit

*AIC BIC logLik p* <

Base -3772.4 -3743.5 1891.2 -

Intercept -3912.0 -3865.7 1964.0 .001

**Slope -3958.6 -3900.8 1989.3 .001**

Quadratic -3953.7 -3878.5 1989.9 *n.s.*

Table 4.

Model comparisons for models fit to the proportion of fixations to the Target (Model 1), Sentence 3-mentioned (Model 2), Semantic Distractor (Model 3), and Sentence 1-mentioned (Model 4) in the Pronoun and Repeated conditions in a 500 ms time window starting at the offset of the critical reference in Sentence 3. Estimates for the base model (no conditions) are also given for comparison. The best fitting model is printed in bold.

*Model 1. Target*

Model Model fit

*AIC BIC logLik p* <

Base -1260.5 -1233.6 635.2 -

**Intercept -1286.0 -1248.3 650.0 .001**

Slope -1284.1 -1241.0 650.0 *n.s.*

Quadratic -1280.6 -1226.8 650.3 *n.s.*

*Model 2. Sentence 3-mentioned*

Model Model fit

*AIC BIC logLik p* <

Base -3091.7 -3064.8 1550.8 -

**Intercept -3097.9 -3060.3 1556.0 .01**

Slope -3098.5 -3055.5 1557.2 *n.s.*

Quadratic -3096.6 -3042.8 1558.3 *n.s.*

*Model 3. Semantic Distractor*

Model Model fit

*AIC BIC logLik p* <

Base -3275.9 -3249.0 1642.9 -

**Intercept -3292.3 -3254.6 1653.1 .05**

Slope -3291.4 -3248.4 1653.7 *n.s.*

Quadratic -3292.6 -3238.8 1656.3 *n.s.*

*Model 4. Sentence 1-mentioned*

Model Model fit

*AIC BIC logLik p* <

Base -2593.2 -2566.3 1301.6 -

Intercept -2646.7 -2609.1 1330.3` .001

**Slope -2649.9 -2606.8 1332.9 .05**

Quadratic -2646.8 -2593.0 1333.4 *n.s.*

Table 5.

Model comparisons for models fit to proportion of fixations to the Semantic Distractor, Sentence 1-unrelated (S1-U), and Sentence 3-mentioned (S3-M) in Sentence 1 in a 500 ms time window starting 100 ms before the offset of the Target in Sentence 1. Estimates for the base model (no conditions) are also given for comparison. The best fitting model is printed in bold.

Model Model fit

*AIC BIC logLik p* <

Base -4175.5 -4145.6 2092.7 -

Intercept -4507.5 -4459.8 2261.8 .001

Slope -4537.4 -4477.8 2278.7 .001

**Quadratic -4547.1 -4469.6 2286.6 .01**

Table 6.

Model comparisons for models fit to the proportion of fixations to the item mentioned with the Target in Sentence 1 in the Repeated (Model 1) and the Pronoun (Model 2) conditions in a 500 ms time window starting at the offset of the critical reference in Sentence 3. Estimates for the base model (no conditions) are also given for comparison. The best fitting model is printed in bold.

*Model 1. Repeated*

Model Model fit

*AIC BIC logLik p* <

Base -3007.8 -2980.0 1508.9 -

**Intercept -3030.5 -2991.6 1522.2 .001**

Slope -3028.6 -2984.1 1522.3 *n.s.*

Quadratic -3027.2 -2971.6 1523.6 *n.s.*

*Model 2. Pronoun*

Model Model fit

*AIC BIC logLik p* <

Base -3148.9 -3121.1 1579.5 -

Intercept -3148.4 -3109.5 1581.2 .18 (*n.s*.)

Slope -3146.6 -3102.2 1581.3 *n.s.*

Quadratic -3142.9 -3087.3 1581.4 *n.s.*

Table 7.

Model comparisons for models fit to examine the difference between the processing of Repeated (Model 1) and Pronoun (Model 2) references in Sentence 3 when Sentence 1 was in the Unrelated versus Related condition in a 500 ms time window starting at the offset of the critical reference in Sentence 3. Proportion of fixations to the (a) Target, (b) Sentence 3-mentioned, (c) Semantic Distractor, and (d) Sentence 1-unrelated served as the outcomes. Estimates for the base model (no conditions) are also given for comparison. The chosen model is printed in bold.

*Model 1. Repeated*

*a. Target*

Model Model fit

AIC BIC logLik p <

Base -1145.3 -1117.5 577.6 -

Intercept -1165.6 -1126.7 589.8 .001

Slope -1172.6 -1128.1 594.3 .01

**Quadratic -1177.4 -1121.8 598.6 .05**

*b. Sentence 3-Mentioned*

Model Model fit

AIC BIC logLik p <

Base -2136.2 -2108.4 1073.1 -

Intercept -2140.5 -2101.6 1077.2 .05

Slope -2138.5 -2094.1 1077.3 *n.s.*

**Quadratic -2148.0 -2092.4 1084.0 .01**

*c. Semantic Distractor*

Model Model fit

AIC BIC logLik p <

Base -2699.1 -2671.3 1354.5 -

Intercept -2697.3 -2658.4 1355.7 *n.s.*

**Slope -2707.1 -2662.6 1361.6 .001**

Quadratic -2704.5 -2637.8 1362.3 *n.s.*

*d. Sentence 1-Unrelated*

Model Model fit

AIC BIC logLik p <

Base -3552.8 -3525.0 1781.4 -

**Intercept -3571.0 -3532.1 1792.5 .001**

Slope -3572.1 -3527.7 1794.1 *n.s.*

Quadratic -3570.9 -3515.3 1795.5 *n.s.*

*Model 2. Pronoun*

*a. Target*

Model Model fit

AIC BIC logLik p <

Base -1553.3 -1525.5 781.7 -

**Intercept -1556.8 -1517.8 785.4 .05**

Slope -1555.3 -1510.8 785.6 *n.s.*

Quadratic -1555.4 -1499.8 787.7 *n.s.*

*b. Sentence 3-mentioned*

Model Model fit

AIC BIC logLik p <

Base -2242.6 -2214.8 1126.3 -

Intercept -2294.1 -2255.2 1154.0 .001

Slope -2299.7 -2255.2 1157.8 .01

**Quadratic -2301.8 -2246.1 1160.9 .05**

*c. Semantic Distractor*

Model Model fit

AIC BIC logLik p <

Base -3349.1 -3321.3 1679.5

Intercept -3369.9 -3331.0 1692.0 .001

**Slope -3375.5 -3331.1 1695.8 .01**

Quadratic -3371.6 -3316.0 1695.8 *n.s.*

*d. Sentence 1-unrelated*

Model Model fit

AIC BIC logLik p <

Base -4917.4 -4869.7 2466.7 -

Intercept -4915.6 -4861.9 2466.8 *n.s.*

Slope -4913.7 -4854.0 2466.8 0.14 (*n.s*.)

Quadratic -4915.3 -4849.6 2468.6 *n.s.*
